# Cosmeceutical Potential of Matcha (*Camellia sinensis*) and Yerba Mate (*Ilex paraguariensis*) Extracts Obtained with Green Solvents

**DOI:** 10.3390/molecules31162742

**Published:** 2026-08-07

**Authors:** Katarzyna Gaweł-Bęben, Monika Szczepanik, Paweł Szczeblewski, Karolina Czech, Wirginia Kukula-Koch

**Affiliations:** 1 Department of Cosmetology, University of Information Technology and Management in Rzeszów, Sucharskiego 2, 35-225 Rzeszów, Poland; monika_kamila_szczepanik@wp.pl (M.S.); kczech@wsiz.edu.pl (K.C.); 2 Department of Pharmaceutical Technology and Biochemistry and BioTechMed Centre, Faculty of Chemistry, Gdansk University of Technology, Gabriela Narutowicza Str. 11/12, 80-233 Gdańsk, Poland; pawel.szczeblewski@pg.edu.pl; 3 Department of Pharmacognosy with Medicinal Plants Garden, Medical University of Lublin, Chodźki 1, 20-093 Lublin, Poland; virginia.kukula@gmail.com

**Keywords:** *Camellia sinensis*, *Ilex paraguariensis*, tyrosinase, antioxidant activity, fibroblasts, melanoma, sun protection factor

## Abstract

Matcha (*Camellia sinensis*) and yerba mate (*Ilex paraguariensis*) are polyphenol-rich plants of growing interest for cosmetic applications, yet the influence of green extraction solvents on their cosmeceutical potential remains underexplored. In this study, aqueous, 20% (*v*/*v*) ethanol (EtOH) and 50% (*v*/*v*) propylene glycol (PG) extracts of both raw materials were prepared by ultrasound-assisted extraction and compared in terms of total phenolic and flavonoid content, antioxidant activity (DPPH and ABTS), tyrosinase inhibition, in vitro sun protection factor (SPF) and cytotoxicity towards human BJ fibroblasts and A375 melanoma cells. Phytochemical profiles were characterized by HPLC-ESI-QTOF-MS/MS, and phytochemistry–activity relationships were explored using hierarchical clustering, Czekanowski analysis and PCA-based regression modeling with leave-one-out cross-validation. Both botanical origin and extraction solvent markedly shaped the composition and biological activity of the extracts, and neither was universally optimal. The matcha 50% PG extract emerged as a promising ready-to-use extract for brightening- and photoprotection-oriented applications, combining strong tyrosinase inhibition with the highest in vitro SPF among the matcha preparations, whereas aqueous and ethanolic extracts showed stronger DPPH scavenging and the most favorable fibroblast safety. For yerba mate, the aqueous and ethanolic extracts were more cytotoxic towards A375 melanoma cells than the PG extract, suggesting a distinct application niche. Chemometric modeling linked biological responses to multivariate phytochemical patterns rather than single compounds, providing marker sets to guide green-solvent extract optimization and cosmetic formulation.

## 1. Introduction

The skin is continuously exposed to ultraviolet (UV) radiation and atmospheric pollutants, which drive excessive reactive oxygen species (ROS) generation. When ROS production exceeds the capacity of the endogenous antioxidant defense system, the resulting oxidative stress contributes to photoaging, hyperpigmentation disorders and an increased risk of cutaneous malignancies, including melanoma [1,2]. Polyphenol-rich plant extracts are among the most intensively investigated multifunctional skin-protecting ingredients, combining radical-scavenging, anti-melanogenic and photoprotective activity within a single extract [3].

*Camellia sinensis* (L.) Kuntze, processed as powdered matcha, represents a specific chemotype of green tea. The tencha leaves used for matcha production are shaded during the final weeks before harvest, which leads to the accumulation of chlorophyll, L-theanine and catechins, including epigallocatechin gallate (EGCG), considered the principal bioactive marker of the raw material [4,5]. EGCG displays a particularly potent radical-scavenging activity (ABTS IC_50_ in the low-µM range), upregulates superoxide dismutase and glutathione peroxidase in fibroblasts exposed to UVB, attenuates MAPK/NF-κB activation and inhibits melanogenesis [5,6]. Although the photoprotective and anti-inflammatory profiles of matcha catechins have been extensively documented, the majority of published studies rely either on purified EGCG or on methanolic, acetonic or hydroalcoholic extracts containing residual organic solvents that are not directly compatible with cosmetic formulation requirements.

*Ilex paraguariensis* A. St.-Hil. (yerba mate) exhibits a qualitatively distinct polyphenolic profile in which caffeoylquinic acid derivatives (mono-, di- and tricaffeoylquinic acids), chlorogenic acid, rutin and kaempferol derivatives dominate, accompanied by methylxanthines, mainly caffeine and theobromine. Phytochemical analyses of commercial yerba mate samples have led to the identification of more than 50 polyphenolic constituents, of which hydroxycinnamic acid derivatives account for approximately 90% of total phenols and flavonols for the remaining 10% [7]. Topical formulations containing *I. paraguariensis* extract have been shown to modulate metalloproteinase and myeloperoxidase activity and to mitigate oxidative damage in murine skin exposed to UVB radiation, supporting both photoprotective and anti-aging applications [8]. Despite the presence of *I. paraguariensis* in INCI lists of cosmetic ingredients, quantitative comparisons of yerba mate with the more thoroughly characterized *Camellia sinensis* extracts—performed under harmonized extraction and biological-screening conditions—remain fragmentary.

In parallel, the cosmetic industry is currently subject to strong regulatory and consumer-driven pressure towards green chemistry: methanol, acetone and concentrated alcohols are progressively being replaced by low-toxicity solvents that are biocompatible with the skin and aligned with the principles of the green chemistry and CHEM21 frameworks [9,10]. The CHEM21 solvent selection guide ranks water, ethanol and propylene glycol (PG) among the recommended solvents on the basis of their combined safety (S), health (H) and environment (E) scores [9]. Beyond their favorable SHE profile, all three solvents simultaneously fulfill functional roles in finished cosmetic products—humectants, co-solvents, preservative boosters and, in the case of PG, penetration enhancers facilitating partitioning of active ingredients into the stratum corneum. Extracts prepared in 50% PG offer the additional advantage of being ready-to-use raw materials: they can be incorporated directly into the aqueous phase of emulsions, tonics or serums without the need for solvent evaporation, which is critical for thermolabile catechins and chlorogenic acid derivatives [11]. Although propylene glycol has occasionally been used as an extraction medium for individual botanical extracts in cosmetic-oriented studies, comprehensive untargeted metabolomic profiling of PG-based plant extracts remains scarce, and, to our knowledge, no study has combined LC-MS-based phytochemical fingerprinting with multivariate correlation to biological activity for extracts prepared in this solvent. So far, however, the systematic LC-MS characterization and biological profiling of matcha and yerba mate extracts in PG-based media has not, to the best of our knowledge, been reported, leaving open both the compositional identity of PG extracts relative to their aqueous and ethanolic counterparts, and the extent to which solvent-dependent shifts in the phytochemical profile translate into measurable differences in antioxidant, anti-melanogenic, photoprotective and cytotoxic activity.

The choice of the three extraction systems used in the present study—water, 20% (*v*/*v*) ethanol and 50% (*v*/*v*) PG—was guided by three converging criteria. First, the extraction properties of the three solvents: water, with its very high hydrogen-bonding component, is selective for hydrophilic glycosylated flavonoids and the most polar caffeoylquinic acid derivatives. Aqueous ethanol (20%, *v*/*v*) introduces an intermediate dispersion–polar–hydrogen-bonding balance favorable for catechins, EGCG and aglycones, whereas 50% PG combines hydrogen-bonding capacity with sufficient dispersive interactions to mobilize weakly polar phenolics and to stabilize oxidation-prone polyphenols through reduced water activity. Second, from a regulatory and toxicological perspective, all three solvents are CHEM21-recommended, and routinely employed in cosmetic formulations, allowing the resulting extracts to be transferred directly to product development without solvent removal [9]. Third, from an industrial standpoint, the three media represent realistic options for cosmetic raw-material manufacturing: aqueous infusion mimics the simplest “tea-like” production protocol, hydroethanolic extraction is currently the standard in the natural-cosmetics segment, and PG-based extraction is increasingly adopted as a green alternative for ready-to-use ingredients with extended microbial stability [11]. Comparing extracts obtained in these three solvents thus allows us to disentangle the contribution of solvent polarity from that of plant chemotype to each measured biological response.

Accordingly, the aim of the present work was to perform a parallel, harmonized characterization of six extracts—matcha (*C. sinensis*) and yerba mate (*I. paraguariensis*) prepared in water, 20% (*v*/*v*) ethanol and 50% (*v*/*v*) PG—by combining: (i) determination of total polyphenol and flavonoid contents; (ii) untargeted metabolomic profiling by LC-MS; (iii) evaluation of antioxidant activity using DPPH and ABTS assays; (iv) assessment of mushroom tyrosinase inhibition; (v) determination of in vitro SPF by the Mansur method; and (vi) evaluation of cytotoxicity towards normal human BJ fibroblasts and A375 melanoma cells. The novelty of the proposed approach lies, first, in the direct head-to-head comparison of two qualitatively distinct polyphenolic chemotypes: catechin-dominated matcha versus caffeoylquinic-acid-dominated yerba mate, under identical analytical and biological conditions, which removes the methodological inconsistencies that typically hamper meta-analyses across the existing literature. Second, we provide, to the best of our knowledge, the first systematic LC-MS characterization of matcha and yerba mate extracts obtained in 50% PG as a ready-to-use cosmetic medium. Third, the data were integrated to identify multivariate phytochemical patterns associated with the measured antioxidant, depigmenting, photoprotective and cytotoxic responses. The results provide a phytochemistry-driven rationale for the design of multifunctional anti-aging, brightening and photoprotection-supporting cosmetic formulations based on solvents compatible with the green-chemistry paradigm.

## 2. Results and Discussion

### 2.1. Comparison of Total Polyphenols, Flavonoids and Antioxidant Activity

The content of polyphenolic compounds in matcha (*C. sinensis*) extracts prepared in different solvents was comparable and ranged from 1.28 to 1.37 mg GAE/mL of extract. In contrast, the 50% PG extract contained twice as many flavonoids (0.31 mg QE/mL) as the remaining extracts. Interestingly, the 50% PG extract showed the weakest antioxidant activity in the DPPH radical scavenging assay (EC_50_ = 0.31% *v*/*v*), but exhibited the strongest reduction in the ABTS radical (EC_50_ < 0.02% *v*/*v*). The 20% EtOH extract, on the other hand, displayed similarly high antioxidant activity in both assays (EC_50_ = 0.04% *v*/*v*). Yerba mate (*I. paraguariensis*) extracts contained a slightly higher polyphenol content than the matcha extracts (1.43–1.54 mg GAE/mL of extract). Again, no statistically significant differences were observed between the solvents used. The flavonoid content in the 20% EtOH extract was significantly lower than in the H_2_O and 50% PG extracts. As with the matcha extracts_,_ the H_2_O and 20% EtOH extracts exhibited comparable antioxidant activity in both the ABTS and DPPH radical scavenging assays. The 50% PG extract, in turn, showed the lowest activity in the DPPH assay (EC_50_ = 0.67% *v*/*v*) and the highest activity in the ABTS assay (EC_50_ = 0.06% *v*/*v*) (Table 1).

The scientific literature reports values of TPC, TFC and antioxidant properties predominantly for green tea infusions rather than solvent extracts. Jakubczyk et al. compared the polyphenol and flavonoid contents in infusions prepared from 11 varieties of green tea available on the Polish market, reporting TPC values in the range of 671.29–1083.13 mg GAE/L and TFC values of 352.36–1359.70 mg rutin equivalents/L, depending on the country of origin, harvest time and cultivation method (conventional vs. organic) [12]. Koch et al. compared the TPC of *C. sinensis* extracts prepared using different extraction methods (accelerated solvent extraction [ASE], ultrasound-assisted extraction [UAE], maceration) and various solvents, including water and a water-ethanol mixture (1:1, *v*/*v*), demonstrating that the hydroethanolic extract contained a higher polyphenol content than the aqueous one (829.89 mg GAE/L vs. 631.76 mg GAE/L). The remaining solvents tested (ethanol, methanol, ethyl acetate, acetone:water mixtures) proved less effective in polyphenol recovery [13]. When expressed on the same volumetric basis, the TPC values obtained for the matcha extracts in the present study (1.28–1.37 mg GAE/mL, corresponding to 1280–1370 mg GAE/L) fall in the same order of magnitude as, and slightly exceed, the ranges reported by Jakubczyk et al. and Koch et al. (631.76–1083.13 mg GAE/L). This is consistent with the higher solid-to-solvent ratio and the use of finely powdered raw material (matcha) employed in this study compared with conventional tea infusions or coarser plant material used in the cited works.

Meanwhile, Martinović et al. compared the content of bioactive compounds and the antioxidant properties of *C. sinensis* extracts obtained in distilled water, 50% ethanol, and selected natural deep eutectic solvents (NaDES): betaine + urea, malic acid + glycerol, tartaric acid + sorbitol, and citric acid + sorbitol, prepared by ultrasound-assisted extraction at 50 °C. In that study, the TPC of the aqueous extract was approximately 60 mg GAE/g DW and that of the ethanolic extract approximately 90 mg GAE/g DW, whereas most of the tested NaDES extracts exhibited even higher TPC values (>100 mg GAE/g DW). This suggests that solvents other than the traditionally used water or aqueous ethanol mixtures are more effective in extracting bioactive compounds from *C. sinensis* leaves. The higher TPC values also translated into higher antioxidant activity of the NaDES extracts compared with the conventional ones [14].

As in the case of matcha, most of the available data on TPC and antioxidant properties of yerba mate also originate from studies on aqueous extracts. Płatkiewicz et al. compared these parameters between cold and hot infusions of 13 yerba mate samples of different origin, commercially available on the Polish market. Higher TPC values (0.900–3.959 mg GAE/mL) and higher antioxidant activity in both the DPPH (0.973–4.776 mg Trolox/mL) and ABTS (0.841–4.079 mg Trolox/mL) radical scavenging assays were detected for cold water extracts [15]. Studies analysing the efficiency of yerba mate extraction using water-ethanol solvents, on the other hand, focus primarily on the quantification of specific compounds. For instance, dos Santos et al. demonstrated that extraction with 50% ethanol resulted in the highest recovery of chlorogenic acid isomers, rutin and *p*-coumaric acid. Similarly, Pilatti-Riccio et al. showed that a 50% water-ethanol extract contained a higher TPC than extracts obtained using pure water or pure ethanol [16].

The discrepancy in antioxidant activity measured by the DPPH and ABTS assays for the 50% PG extracts of both investigated plant materials can be explained by well-documented differences in the interaction mechanisms of these radicals with various types of antioxidants. ABTS, being a more hydrophilic molecule and smaller in size than DPPH, reacts more effectively with hydrophilic antioxidants such as phenolic acids, flavonoid glycosides and ascorbic acid, including those of higher molecular weight. DPPH, by contrast, is more efficiently reduced by lipophilic molecules of smaller structure, which have better access to the radical center [17,18].

### 2.2. Comparison of the Skin-Lightening Potential

The skin-lightening potential of the matcha and yerba mate extracts was subsequently compared by assessing their effect on the activity of tyrosinase (EC 1.14.18.1), a copper-containing oxidoreductase that catalyzes the rate-limiting step of melanin biosynthesis—the hydroxylation of L-tyrosine to L-DOPA and its subsequent oxidation to dopaquinone [19]. The inhibition of melanogenesis through the modulation of tyrosinase activity, as well as of other factors regulating melanin biosynthesis, has been well documented in the literature for matcha extracts. Several catechins present in tea have been identified as melanogenesis inhibitors [20,21], and recent studies have additionally shown the influence of amino acids (L-theanine) and tea-derived peptides on melanogenesis [22,23].

**Figure 1:**
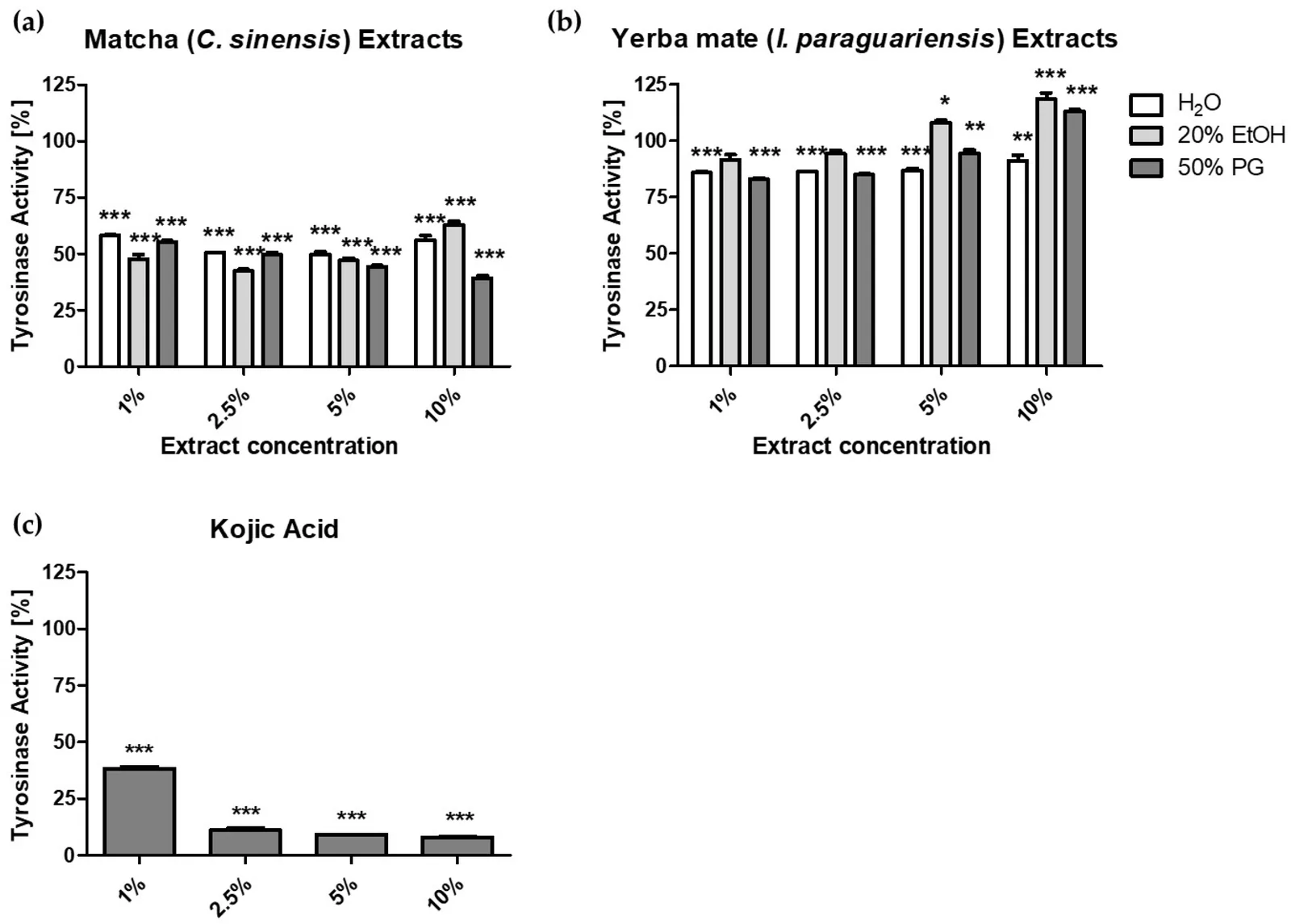
Comparative analysis of the effect of (**a**) matcha and (**b**) yerba mate extracts on tyrosinase activity. Kojic acid at similar concentrations was used as positive control (**c**). Data are presented as mean ± SD; *n* = 3. Statistically significant differences are indicated as follows: * *p* < 0.05, ** *p* < 0.01; *** *p* < 0.001.

The effectiveness of matcha extracts in inhibiting tyrosinase was also confirmed in the present study (Figure 1a), where all the analyzed extracts at concentrations of 1–10% (*v*/*v*) inhibited tyrosinase activity by 37–61%. The strongest inhibition was observed for the 50% PG extract, which at a concentration of 10% (*v*/*v*) reduced tyrosinase activity to ca. 39%.

For comparison, the yerba mate extracts exhibited markedly lower activity against tyrosinase, inhibiting the enzyme by less than 20% (Figure 1b). Yerba mate extracts have not been previously described in the literature as effective inhibitors of tyrosinase or of other steps of melanogenesis. Kuijpers et al. demonstrated that a yerba mate extract—specifically, the chlorogenic-acid-rich phenolic fraction—inhibits the activity of tyrosinase from *Agaricus bisporus* (mushroom tyrosinase) while, in contrast, activating tyrosinase isolated from potato [24].

### 2.3. Comparison of the Sun Protecting Potential

The next stage of the comparative study involved the assessment of the photoprotective potential of the prepared extracts using the Mansur method [25]. The Mansur spectrophotometric method provides an in vitro estimate of SPF based on UV absorbance measurements in solution and is primarily sensitive to the UVB range (290–320 nm). In the present study, extracts from *C. sinensis* demonstrated significantly lower SPF values than extracts from *I. paraguariensis* (Table 2). Among *C. sinensis* extracts, the highest in vitro SPF was recorded for the 50% PG extract—at a concentration of 5%, it reached 18.54. In turn, the in vitro SPF values for *I. paraguariensis* extracts at concentrations of 2.5% and 5% exceeded 20, with the highest values observed for the H_2_O and 50% PG extracts. At the lowest tested concentration (1%), the highest SPF was recorded for the 20% EtOH extract, reaching 18.12 (Table 2).

Since the Mansur equation considers only the UVB range (290–320 nm), full UV absorption spectra (200–400 nm) were additionally recorded for all extracts to evaluate their spectral quality across the UVB and UVA regions (Figures S5 and S6). From these spectra, the critical wavelength (λc) and the ratio of UVA (320–400 nm) to UVB (290–320 nm) absorbance were calculated for each extract (Table S6). Matcha extracts reached λc values of 371.7–380.7 nm across all tested solvents and concentrations, meeting the conventional λc ≥ 370 nm threshold used to classify broad-spectrum UVB/UVA protection. Yerba mate extracts, in contrast, showed lower λc values (350.4–364.3 nm), indicating a narrower spectral range weighted more heavily towards UVB, despite displaying higher UVA/UVB absorbance ratios (up to 1.90) than matcha extracts (up to 1.34). These findings suggest that, although yerba mate extracts contribute more strongly to UVA absorbance in relative terms, matcha extracts provide a more spectrally balanced UV-absorption profile consistent with broad-spectrum classification criteria.

It should be emphasized that the Mansur equation provides only a spectrophotometric estimate of UVB-absorbing capacity (290–320 nm) for extracts diluted in solution, and does not correspond to the official in vitro (ISO 23675) or in vivo (ISO 24444) SPF determination methods, nor does it capture UVA protection (ISO 24443) or photostability [26,27,28]. In particular, catechins such as EGCG, the principal bioactive markers of matcha, are known to be photounstable upon prolonged UV exposure, with reported losses of up to 77–85% after 1 h of simulated solar irradiation in topical formulations [29], and the single-timepoint absorbance measurement used in the Mansur method does not capture any pre-/post-irradiation change in UV-absorbing capacity, unlike the ISO 24443 protocol.

Unlike standardized SPF testing, which evaluates a complete formulation applied at a defined density (2 mg/cm^2^ in vivo or 1.3 mg/cm^2^ on a PMMA plate in vitro), the present measurements were performed on the extracts alone in solution, at concentrations (1–5%) representative of typical cosmetic incorporation rates but outside the context of a finished formula. Despite these limitations, the Mansur method remains a rapid, low-cost and widely used screening tool that allows relative ranking of candidate extracts and solvents at an early formulation-development stage, before committing to more resource-intensive standardized testing, as demonstrated by comparative screenings of multiple plant extracts and extraction solvents for their in vitro UVB-absorbing potential [30,31]. The reported SPF values should therefore be interpreted as an indication of intrinsic UVB-absorbing potential and a possible SPF-boosting contribution when incorporated into a sunscreen formulation, rather than as a validated photoprotective efficacy claim.

The photoprotective properties of extracts from *C. sinensis* and *I. paraguariensis* have previously been investigated, with the photoprotective potential of *C. sinensis* and its bioactive compounds being considerably better documented in the scientific literature. Pasupathi et al. demonstrated the SPF-enhancing effect of green tea extract. A formulation containing 5% green tea extract in combination with the synthetic UV filter octocrylene exhibited a higher in vitro SPF than octocrylene alone [32]. The same research group further demonstrated that green tea extracts can be employed in the green synthesis of ZnO nanoparticles (ZnO NPs), which also function as SPF boosters. Formulations containing ZnO NPs and octocrylene exhibited in vitro SPF values that were 1.4–1.65-fold higher than those of octocrylene alone and 3.7–4.5-fold higher than those of ZnO NPs alone [33]. In a recent study, Said et al. also demonstrated the photoprotective efficacy of a hydrogel formulated with green tea extract [34].

In contrast, evidence supporting the photoprotective potential of *I. paraguariensis* is limited. To date, only one study has reported the photoprotective activity of a hydroethanolic leaf extract of *I. paraguariensis*. Incorporation of the extract into a cosmetic formulation reduced the activity of matrix metalloproteinases and myeloperoxidase in the skin of UVB-exposed mice, suggesting a protective effect against UV-induced skin damage [8]. To the best of our knowledge, these are the only published data demonstrating the photoprotective potential of *I. paraguariensis* extracts.

### 2.4. Comparison of In Vitro Cytotoxicity and Anti-Melanoma Potential

**Figure 2:**
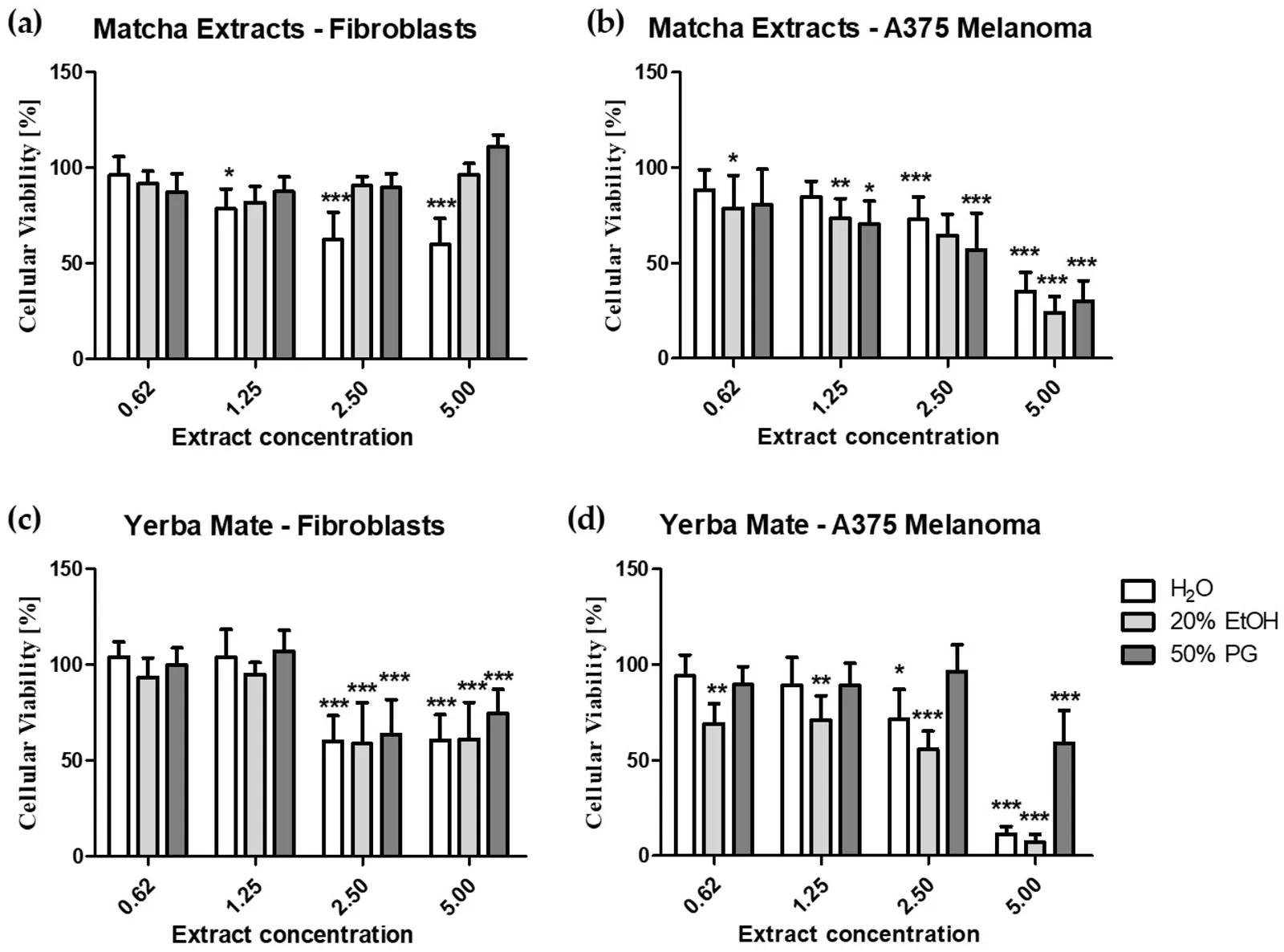
Cytotoxic effect of matcha (**a**,**b**) and yerba mate (**c**,**d**) extracts on human fibroblasts (**a**,**c**) and A375 melanoma cells (**b**,**d**) following 48 h of treatment. Data are presented as mean ± SD of three independent experiments (*n* = 3), each performed with five technical replicates per concentration. Statistically significant differences are indicated as follows: * *p* < 0.05; ** *p* < 0.01; *** *p* < 0.001. To quantitatively assess the selectivity of the cytotoxic activity of tested extracts, IC_50_ values were calculated for both A375 melanoma and BJ fibroblast cell lines, and the Selectivity Index (SI) was determined for each extract (Table 3). All aqueous and ethanolic extracts of both matcha and yerba mate exhibited IC_50_ values against A375 cells in the range of 2.71–3.81%, whereas BJ fibroblast viability remained above 50% across the entire tested concentration range (IC_50_ > 10%), yielding SI values between 2.62 and 3.69, consistent with preferential cytotoxicity towards melanoma cells over fibroblasts. The 50% PG extract of matcha showed a comparable IC_50_ against A375 cells (2.99%) and an equally favorable SI (>3.35). In contrast, the 50% PG extract of yerba mate did not reduce A375 viability below 50% within the tested concentration range (IC_50_ > 10%), similarly to its effect on BJ fibroblasts; consequently, the SI could not be determined for this extract, and no conclusion regarding its selective cytotoxic potential can be drawn from the present data.

Assessment of the cytotoxicity of plant extracts towards skin cells is a critical step in validating their cosmetic and dermatological potential. An ideal active ingredient should exhibit a “dual profile”: the absence of cytotoxicity towards normal skin cells (keratinocytes, fibroblasts) at concentrations with documented functional activity, combined with selective cytotoxicity towards abnormal or neoplastic cells (e.g., melanoma cell lines). Extracts from both analysed raw materials demonstrated precisely this type of selectivity. Among the matcha extracts, only the H_2_O extract significantly reduced fibroblast viability at the highest tested concentrations (2.5% and 5%); however, the reduction in viability did not exceed 50% (Figure 2a). In contrast, all analysed matcha extracts reduced the viability of A375 melanoma cells below 50% (Figure 2b). In the case of *I. paraguariensis*, all extracts at concentrations of 2.5% and 5% reduced fibroblast viability to approximately 55–65% (Figure 2c), whereas the H_2_O and 20% EtOH extracts proved most cytotoxic towards A375 cells, reducing the percentage of viable cells below 20% at the highest tested concentration (Figure 2d).

**Table 3:** Half-maximal inhibitory concentration (IC_50_) against A375 melanoma and BJ fibroblast cell lines, and the corresponding Selectivity Index (SI), for matcha (*C. sinensis*) and yerba mate (*I. paraguariensis*) extracts prepared in water (H_2_O), 20% (*v*/*v*) ethanol (20% EtOH) and 50% (*v*/*v*) propylene glycol (50% PG); SI = IC_50_(BJ_50_/IC_50_(A375); n.d. = not determinable, as neither cell line reached 50% viability within the tested range.

Green Solvent	IC_50_ A375 Melanoma (%, *v*/*v*)	IC_50_ BJ Fibroblasts (%, *v*/*v*)	Selectivity Index (SI)
Matcha *(Camellia sinensis)* extracts			
H_2_O	3.81	>10	>2.62
20% EtOH	3.21	>10	>3.12
50% PG	2.99	>10	>3.35
Yerba mate *(Ilex paraguariensis)* extracts			
H_2_O	3.20	>10	>3.13
20% EtOH	2.71	>10	>3.69
50% PG	>10	>10	n.d.

The scientific literature on the cytotoxicity of both investigated species is characterised by a marked asymmetry. *C. sinensis* and its principal bioactive constituent EGCG have been extensively studied across multiple skin cell line types, with documented absence of toxicity towards normal cells [35] and selective cytotoxicity towards melanoma cell lines [36,37]. The present study demonstrates that the selection of an appropriate extraction solvent may favorably influence the selective cytotoxicity of *C. sinensis* extracts towards melanoma cells.

*I. paraguariensis, however,* remains markedly understudied in this regard—only one publication is available demonstrating the absence of cytotoxicity of fermented yerba mate extracts towards keratinocytes and fibroblasts under in vitro conditions [38]. The results presented in this study therefore contribute to filling the research gap concerning the effect of *I. paraguariensis* extracts on the viability of keratinocytes and melanoma cells.

### 2.5. Comparison of the Phytochemical Composition

High-performance liquid chromatography coupled with mass spectrometry (HPLC-ESI-QTOF-MS/MS) analysis of *C. sinensis* and *I. paraguariensis* extracts led to recording clear fingerprints with successfully separated peaks in both ionization modes. The performed compounds’ assignment that was done with help of the scientific literature and commercially available mass databases led to the tentative identification of various specialised metabolites (see Table 4 and Table 5 and Figures S1–S4 and Table S1).

The performed analyses revealed distinct solvent-dependent differences in the relative abundance of their major bioactive constituents. As summarized in Table 4, caffeine was the dominant compound in all matcha extracts, maintaining consistently high relative abundance regardless of the solvent used—consistent with its well-documented hydrophilic character and efficient recovery across a wide range of aqueous and hydroorganic media [39]. Alongside caffeine, caffeoylquinic acid derivatives, theanine and gallocatechin represent the most characteristic and consistently detected constituents of matcha [40] and both appeared as stable components across all three solvent systems in the present study. Matcha extracts were also sources of catechins. Epicatechin, epicatechin gallate (ECG) and epigallocatechin gallate (EGCG) were highly represented in the tested samples, especially in the 20% EtOH and 50% PG preparations. EGCG and epicatechin gallate concentrations are known to be significantly higher in more solvent-rich extracts and remain only residual in purely aqueous preparations [41], which is consistent with prior reports demonstrating that aqueous ethanol mixtures extract significantly more catechins than water alone, owing to the partial disruption of hydrogen-bond networks that facilitates release of moderately polar compounds from the plant cell wall. Given the well-established role of catechin gallates as inhibitors of melanogenesis [42], this selective enrichment more likely accounts for the stronger tyrosinase inhibitory activity observed for the 20% EtOH and 50% PG matcha extracts in Section 2.2.

A broadly similar picture emerged for yerba mate extracts, where caffeine again dominated across all extracts (+++). The primary polyphenols of yerba mate extracts are chlorogenic acids, particularly the 3-, 4-, and 5-caffeoylquinic acid isomers, with rutin and other flavonoids present in smaller quantities [43]. Consistent with this, caffeoylquinic acid derivatives were consistently detected at lower abundance in 50% PG. An additional caffeoyl derivative appeared uniquely in the 20% EtOH preparation, suggesting that solvents of intermediate polarity selectively favour the extraction of certain phenolic acid conjugates—a pattern supported by the observation that water–organic mixtures enhance recovery of less polar polyphenols such as dicaffeoylquinic acids compared to water alone [44].

The advantage of hydroorganic systems over pure water was equally evident for flavonoids in yerba mate extracts as well. Epicatechin, rutin, and kaempferol-*O*-galactoside were not that efficiently detectable in H_2_O extracts, whereas rutin and kaempferol-*O*-galactoside were successfully recovered at low abundance in both the 20% EtOH and 50% PG extracts. However, the 50% PG was found to be an efficient extracting solvent of catechins. The 50% PG yerba mate extract further yielded gallocatechin, epicatechin gallate and epicatechin, pointing to a particular affinity of propylene glycol for catechin-type compounds [45]. In the case of matcha extracts, the ECG, EGCG, and EC were present in the mass chromatograms at higher quantities. Taken together, these patterns reflect the varying polarities of the recovered metabolites and demonstrate that binary hydroorganic mixtures, whether ethanol- or propylene glycol-based, offer considerably broader phytochemical coverage than aqueous extraction alone [44].

### 2.6. Statistical and Chemometric Analysis of Matrix- and Solvent-Dependent Activity Profiles

The statistical and chemometric analyses were used to answer three formulation-relevant questions: whether the observed differences between extracts were reproducible, whether the extracts and phytochemical markers followed common patterns, and which chemical patterns were associated with the measured cosmetic endpoints. Detailed statistical outputs are provided in Supplementary Tables S2–S5.

#### 2.6.1. Effects of Plant Matrix and Extraction Solvent on Biological Activity

**Figure 3:**
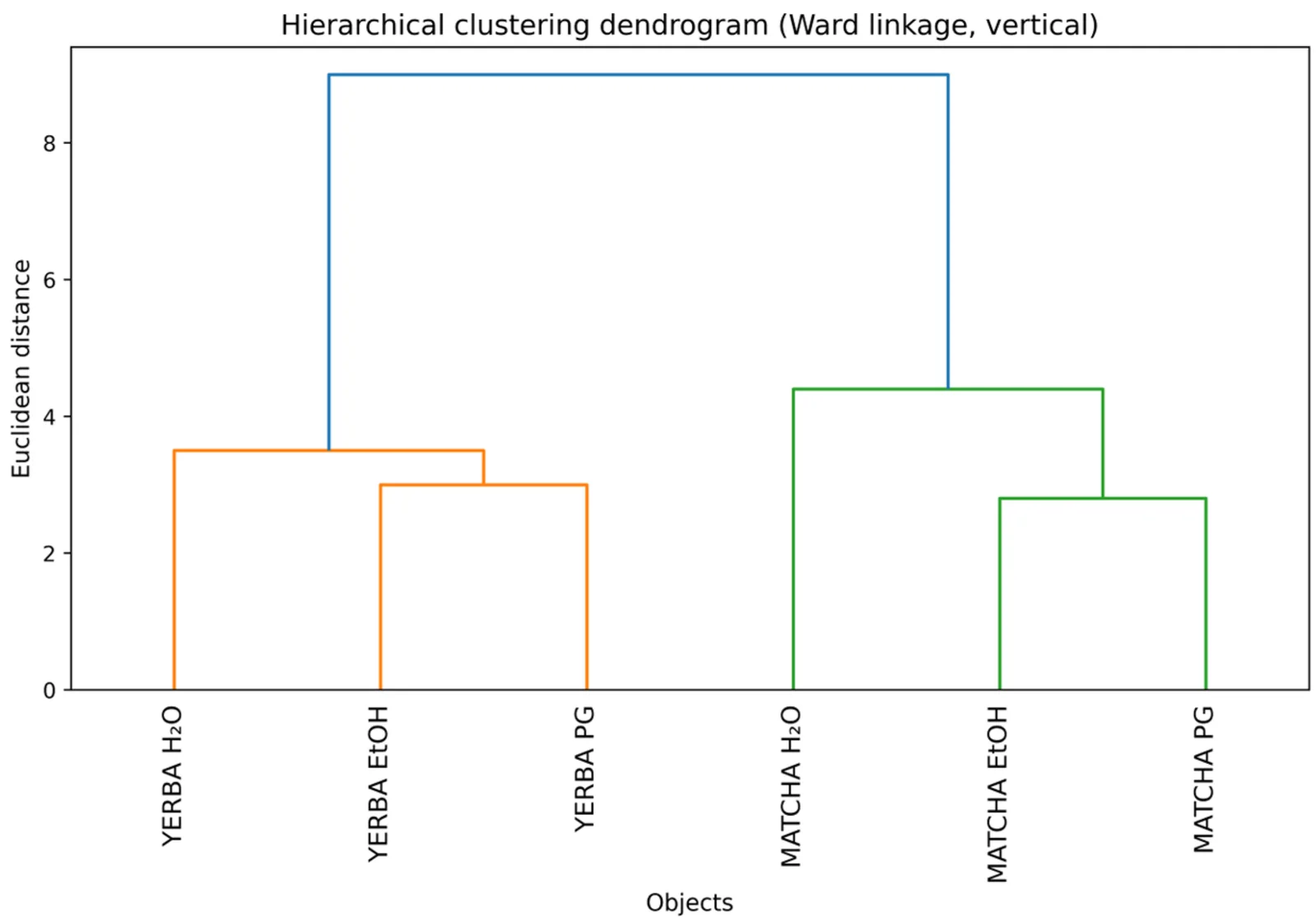
Hierarchical clustering dendrogram of standardized matcha and yerba mate extracts. The dendrogram was calculated from autoscaled phytochemical profiles using Euclidean distance and Ward’s linkage. The separation of matcha and yerba mate indicates that botanical origin was the strongest source of global phytochemical variation, while the closer pairing of 20% EtOH and 50% PG extracts within each matrix indicates solvent-related similarity.

**Figure 4:**
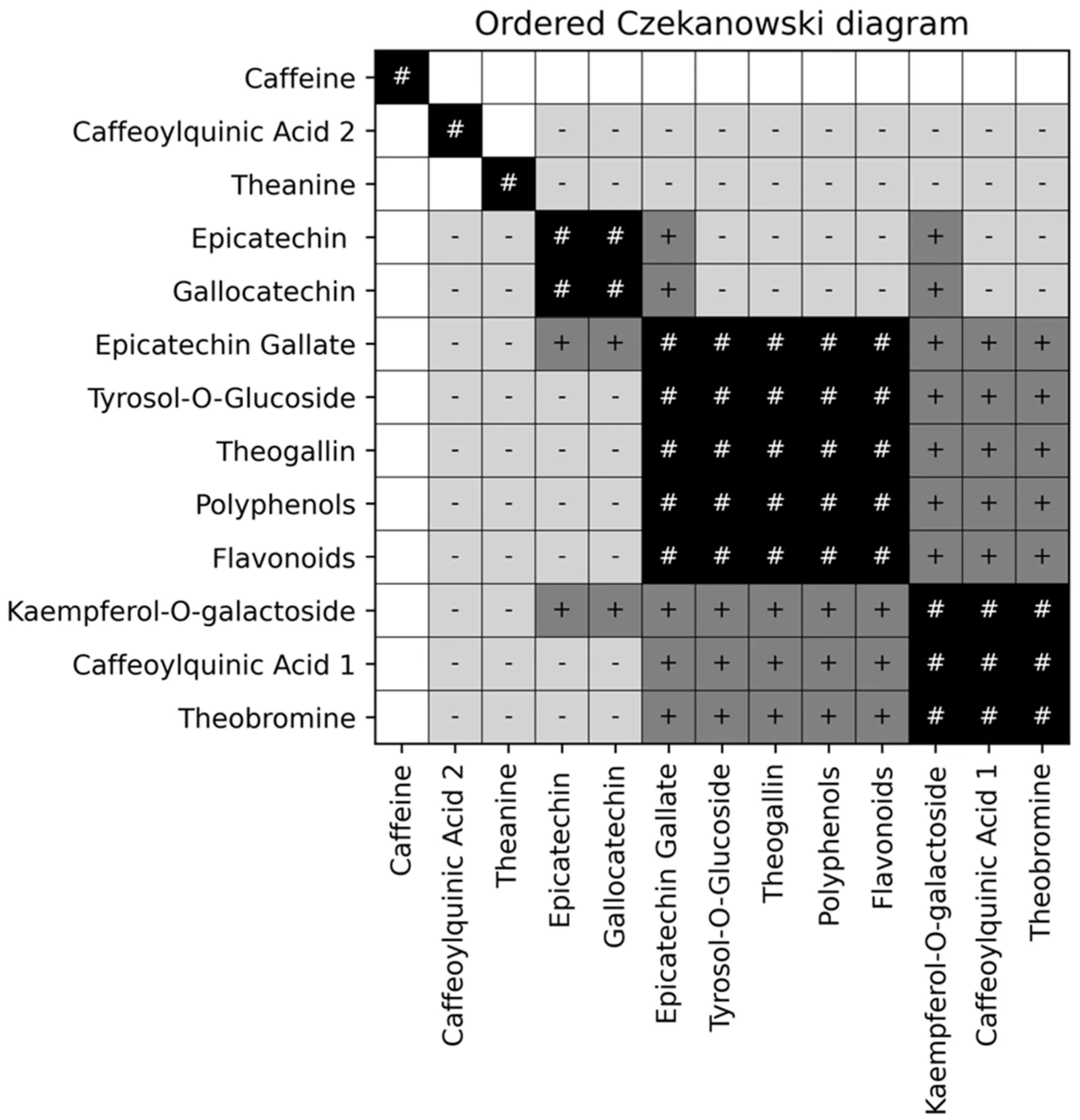
Ordered Czekanowski diagram of phytochemical markers determined in matcha and yerba mate extracts. The diagram was constructed from autoscaled profiles of eleven LC–MS markers together with total polyphenol and total flavonoid contents. Symbols indicate Euclidean-distance classes: “#”, ≤0.5; “+”, >0.5–1.0; “-”, >1.0–1.5; blank, >1.5. The diagram visualizes co-varying marker patterns across the six extracts, not direct causal relationships or absolute concentrations.

Univariate statistics were first used to determine whether the apparent differences between extracts exceeded experimental variability. The detailed ANOVA and Tukey HSD outputs are provided in Supplementary Tables S2 and S3. The results show that neither plant material nor solvent can be considered universally optimal. In matcha, the 50% PG extract provided the highest in vitro SPF and tyrosinase inhibition, whereas aqueous and ethanolic extracts showed stronger DPPH radical-scavenging activity. In yerba mate, the aqueous and ethanolic extracts displayed higher A375 cytotoxicity than the 50% PG extract, while ethanolic extracts were the least cytotoxic toward fibroblasts in both botanical matrices. These endpoint-specific trade-offs provide the statistical context for the compositional relationships subsequently visualized in Figure 3 and Figure 4 and are consistent with the fact that solvent choice changes the balance of recovered phenolics rather than simply increasing total extractability [13].

#### 2.6.2. Hierarchical Clustering and Czekanowski Analysis of Phytochemical Profiles

Hierarchical clustering analysis (HCA) was used as an intuitive overview of the global phytochemical similarity of the six extracts: short branches indicate more similar overall profiles, whereas long branches indicate greater differences. As shown in Figure 3, HCA clearly separated matcha from yerba mate, showing that botanical origin was the main determinant of the phytochemical fingerprint. Within each plant matrix, the 20% EtOH and 50% PG extracts clustered more closely with each other than with the aqueous extract. Thus, 50% PG did not simply reproduce an aqueous infusion; instead, it generated an overall profile closer to the hydroethanolic preparation. This is important for formulation development because 50% PG extracts can be incorporated directly into cosmetic systems, avoiding solvent removal while retaining a phytochemical profile distinct from, but globally related to, the conventional hydroethanolic extract [9,10].

The ordered Czekanowski diagram (Figure 4) complements HCA by showing which measured variables followed similar patterns across the six extracts. It does not rank compounds by absolute concentration or biological potency; rather, it identifies variables that co-varied across the dataset. Epicatechin and gallocatechin formed the closest pair. This is chemically plausible because both are structurally related flavan-3-ols. A second block linked epicatechin gallate, tyrosol-*O*-glucoside, theogallin, total polyphenols and total flavonoids, whereas kaempferol-*O*-galactoside, caffeoylquinic acid 1 and theobromine formed another block. These blocks can be regarded as candidate marker packages for future targeted enrichment and bioassay-guided fractionation, rather than as ready-made causal explanations of activity.

Caffeine was highly abundant in the LC–MS fingerprints of both botanical materials, but it was separated from the other marker blocks in Figure 4. Because the chemometric data were autoscaled, this separation means that caffeine varied differently across the six extracts. Moreover, LC–MS peak areas cannot be compared directly between chemically different analytes as a measure of absolute amount or potency. In the present regression interpretation (Equations (1)–(3) and Table 5), caffeine was associated only with lower fibroblast cytotoxicity, and no reliable model was obtained for A375 cytotoxicity or DPPH scavenging activity. Therefore, its high relative LC–MS signal cannot be used to explain the overall biological profile of the extracts.

#### 2.6.3. PCA-Based Regression Analysis of Phytochemical Patterns Associated with Cosmetic Endpoints

PCA-based regression was used to move from similarity patterns to an exploratory activity-oriented interpretation. In practical terms, the models evaluated which combinations of co-varying markers were associated with a higher or lower responses; they were not intended to identify individual compounds as causal determinants of the observed activities. The PCA loadings and full regression-validation statistics are provided in Supplementary Tables S4 and S5. Three exploratory e models were obtained for tyrosinase inhibition, in vitro SPF and fibroblast cytotoxicity and are expressed in Equations (1)–(3). Leave-one-out cross-validation yielded high internal Q^2^ values for the tyrosinase-inhibition model (Q^2^LOO = 0.976) and the fibroblast-cytotoxicity model (Q^2^LOO = 0.912). However, given small number of extracts, these internal cross-validation results may overestimate model performance and should not be interpreted as evidence of robust predictive ability beyond the present dataset. The SPF model showed more moderate internal cross-validation performance (Q^2^LOO = 0.550) and was therefore considered suitable only for exploratory interpretation. No satisfactory model was obtained for A375 cytotoxicity or DPPH activity, indicating that these endpoints were not adequately captured by the selected linear phytochemical patterns.

Equations (1)–(3). Chemometric models of factor contributions to biological activity. F1–F3 are PCA-derived latent factors; the equations describe multivariate associations within the present dataset.

Tyrosinase inhibition = 0.54 × F1 + 0.48 × F3(1)

Sun protection factor = −1.77 × F1 + 1.06 × F3(2)

Fibroblast cytotoxicity = 0.79 × F1 − 1.58 × F2(3)

As summarized in Table 6 and expressed in Equations (1)–(3), the tyrosinase model associated higher inhibition with a pattern containing theanine, theogallin and tyrosol-*O*-glucoside. This interpretation should be regarded as a direction for marker-guided optimization, not as a replacement for direct testing of the individual compounds. It is nevertheless consistent with the established cosmetic relevance of tea-derived catechins and other polyphenolic constituents for melanogenesis-related pathways [4,5,6,20,21,22]. The SPF model associated higher values with flavonoids, tyrosol-*O*-glucoside and caffeoylquinic-acid features; because its predictive ability was moderate (Supplementary Table S5), this pattern should be used as a formulation-screening cue rather than a quantitative prediction tool. This is compatible with the known UV-absorbing and antioxidant roles of phenolic systems, while recognizing that an in vitro SPF estimate is not a substitute for in vivo sunscreen performance [4,5,6].

For fibroblast cytotoxicity, Equations (1)–(3) and Table 6 associated catechin-related markers and caffeine with lower cytotoxicity, whereas theobromine, theanine, theogallin and caffeoylquinic-acid features were associated with higher cytotoxicity. This does not establish that any individual compound is cytoprotective or cytotoxic. Rather, it identifies a composite marker pattern that can be monitored when prioritizing a safety-oriented cosmetic ingredient. The practical message is that ethanolic extracts provided the most favorable fibroblast-safety profile in the present assay, while the matcha 50% PG extract offered a particularly attractive combination of high tyrosinase inhibition and SPF. 50% PG should therefore be viewed as a selective, formulation-ready green extraction medium rather than as a universal substitute for water or ethanol: it preserved a hydroethanolic-like global profile in Figure 3 and supported selected cosmetic endpoints, but it was less favorable in the DPPH assay and did not maximize every response. Further stability, skin-compatibility and formulation studies are required before selecting a final solvent system for product development.

Taken together, Figure 3 and Figure 4, Equations (1)–(3), and Table 6 provide a coherent formulation narrative. Botanical matrix determines the baseline chemical identity of the extract, solvent adjusts the balance of co-extracted marker groups, and the most suitable extract depends on the intended claim.

However, the PCA-based regression models identify associations only and do not establish causal contributions of individual compounds or compound combinations. In particular, the associations of theanine, theogallin and other markers with tyrosinase inhibition require targeted validation using isolated compounds and defined mixtures. These findings should therefore be regarded as hypotheses for future mechanistic studies and as guidance for extract optimization rather than evidence of compound-specific activity.

#### 2.6.4. Conclusions and Perspectives for Cosmetic Formulation

This study demonstrates that both botanical origin and extraction solvent shape the phytochemical profile and cosmetic potential of matcha and yerba mate extracts. Matcha 50% PG emerged as a promising ready-to-use extract for brightening- and photoprotection-oriented applications, combining high tyrosinase inhibition with the highest in vitro SPF among the matcha preparations. In contrast, aqueous and ethanolic extracts showed stronger DPPH radical-scavenging activity, while ethanolic extracts displayed the most favorable fibroblast-safety profile.

These solvent-dependent differences are likely related, at least in part, to the differing physicochemical properties of the extraction media. Water has a markedly higher dielectric constant and hydrogen-bond-donating/accepting capacity than either aqueous ethanol or aqueous propylene glycol, which favors the extraction of highly polar glycosylated flavonoids and caffeoylquinic acid derivatives, but may limit the release of less polar catechins and aglycones from the plant matrix. The lower dielectric constant of the 20% EtOH and 50% PG systems, together with the amphiphilic, diol-based structure of propylene glycol, could explain the comparatively higher catechin and flavonoid content observed in the corresponding extracts, as well as their distinct DPPH and ABTS activity profiles.

The chemometric analyses further showed that biological responses were associated with phytochemical patterns rather than with the abundance of a single compound, providing practical marker sets for future extract optimization and bioassay-guided formulation development.

## 3. Materials and Methods

### 3.1. Chemicals and Reagents

Human malignant melanoma A375 (ATCC CRL-1619) and BJ fibroblast (ATCC CRL-2522) cell lines were obtained from ATCC via LGC Standards (Łomianki, Poland). Dulbecco’s phosphate-buffered saline (DPBS), Eagle’s Minimum Essential Medium (EMEM), Dulbecco’s Modified Eagle’s Medium (DMEM), 2,2-diphenyl-1-picrylhydrazyl (DPPH), 2,2′-azino-bis(3-ethylbenzothiazoline-6-sulfonic acid) (ABTS), tyrosinase from Agaricus bisporus (≥1000 U/mg, lyophilized powder), 3,4-dihydroxy-L-phenylalanine (L-DOPA), quercetin (≥95% purity), gallic acid (≥97.5% purity), and Neutral Red solution in DPBS (3.3 g/L) were purchased from Sigma-Aldrich (St. Louis, MO, USA). Propylene glycol (PG, ≥99.8% purity), Folin–Ciocalteu reagent, and potassium persulfate (K_2_S_2_O_8_, ≥99% purity) were obtained from Chempur (Piekary Śląskie, Poland). Ethanol (≥99.8% purity) was obtained from Honeywell (Charlotte, NC, USA). Fetal bovine serum (FBS) was obtained from Pan-Biotech (Aidenbach, Germany). The solvents used for the compositional study of the extracts by LC-MS (water, acetonitrile, and formic acid) were purchased from Merck (Darmstadt, Germany).

### 3.2. Plant Material and Extract Preparation

Dried herbs of yerba mate (*Ilex paraguariensis*) cultivated in Brazil and matcha (*Camellia sinensis*) cultivated in China, both obtained from EU-certified organic farming, were purchased from Dary Natury (Koryciny, Poland) and Solid Food (Piaseczno, Poland), respectively. Voucher specimens of both plant materials are deposited at the Department of Cosmetology, University of Information Technology and Management in Rzeszów, Poland. For extract preparation, 3 g of ground plant material was mixed with 100 mL of one of the following solvents (3%, *w*/*v*): ultrapure water (H_2_O), 20% (*v*/*v*) ethanol (EtOH) in water, or 50% (*v*/*v*) propylene glycol (PG) in water, and sonicated for 3 h at room temperature (RT, 22 ± 2 °C) using an ultrasonic bath (Sonic-3, Polsonic, Warsaw, Poland; 40 kHz, 100 W). The obtained extracts were filtered through filter paper (Whatman No. 1) and a 0.45 μm PES sterile syringe filter, and stored at −20 °C until analysis.

### 3.3. Total Polyphenolic Content (TPC)

TPC was determined spectrophotometrically using a modified protocol of Fukumoto and Mazza [46]. Briefly, 150 μL of the undiluted extract was mixed with 750 μL of Folin–Ciocalteu reagent (diluted 1:10 with ultrapure water). After 6 min of incubation at room temperature (RT), 600 μL of 7.5% (*w*/*v*) Na_2_CO_3_ was added. The absorbance of the samples was measured after 2 h of incubation in the dark at λ = 740 nm using a DR6000 UV-Vis spectrophotometer (Hach-Lange, Wrocław, Poland). Calibration curves were prepared using 0–100 μg/mL gallic acid in water (y = 0.0149x − 0.0058; R^2^ = 0.9997), 20% ethanol (y = 0.0145x − 0.0042; R^2^ = 0.9995), and 50% PG (y = 0.0128x − 0.0276; R^2^ = 0.9916). All measurements were performed in triplicate. The content of phenolic compounds is expressed as mg of gallic acid equivalents per mL of the extract (mg GAE/mL).

### 3.4. Total Flavonoid Content (TFC)

TFC was assessed as described by Matejić et al. [47]. Briefly, 150 μL of the undiluted extract was mixed with 645 μL of the reaction mixture (61.5 mL of 96% (*v*/*v*) ethanol, 1.5 mL of 10% (*w*/*v*) Al(NO_3_)_3_, and 1.5 mL of 1 M CH_3_COOK). After 40 min of incubation at RT, the absorbance was measured at λ = 415 nm using a DR6000 UV-Vis spectrophotometer (Hach-Lange, Wrocław, Poland). The concentration of flavonoids in the analyzed extracts was calculated based on quercetin calibration curves prepared in water (*y* = 0.0076*x* + 0.0046; *R*^2^ = 0.9984), 20% ethanol (*y* = 0.0076*x* + 0.0028; *R*^2^ = 0.9991), and 50% PG (*y* = 0.0055*x* + 0.0019; *R*^2^ = 0.9914). All measurements were performed in triplicate. The content of flavonoids was expressed as mg of quercetin equivalents per mL of the extract (mg QE/mL).

### 3.5. DPPH Radical Scavenging Assay

The antioxidant properties of the extracts were determined based on their ability to scavenge the DPPH radical, according to the procedure of Matejić et al. [47] with some modifications. The extracts were serially diluted in the corresponding solvent (water, 20% (*v*/*v*) ethanol, or 50% (*v*/*v*) propylene glycol) at final concentrations ranging from 0.048% to 100% (*v*/*v*). Then, 100 μL of the diluted extract was mixed with 100 μL of 0.1 mM DPPH solution in methanol and incubated in the dark for 20 min at room temperature. For each analyzed extract, the corresponding solvent mixed with the DPPH solution was used as the control. L-ascorbic acid was used as the positive control. The absorbance was measured at λ = 540 nm using a FilterMax F5 microplate reader (Molecular Devices, San Jose, CA, USA). The absorbance values were corrected by subtracting the absorbance of the corresponding sample blank (without DPPH) to account for the intrinsic color of the extracts. The percentage of DPPH radical scavenging activity was calculated according to the following formula:

(4) $$DPPH\;Scavenging\;Activity\left[\%\right]=\left(1-\;\frac{Abs_{S}}{Abs_{S}}\;\;\right)\times 100\%$$

where Abs___S is the absorbance of the sample and Abs_C is the absorbance of the corresponding solvent control. The half-maximal effective concentration (EC_50_) was calculated from the dose–response curves using GraphPad Prism, version 8.4.3 for Windows (GraphPad Software, San Diego, CA, USA). All measurements were performed in triplicate.

### 3.6. ABTS Radical Scavenging Assay

The antioxidant properties of the extracts were tested using the ABTS radical scavenging assay, according to the procedure of Re et al. [48] with some modifications. The ABTS stock solution was prepared by reacting 7 mM ABTS with 2.45 mM K_2_S_2_O_8_ in the dark for 16 h at room temperature, then diluted with ultrapure water to obtain a working solution with an absorbance of 1.0 ± 0.02 at 405 nm. Then, 15 μL of the extract diluted in the corresponding solvent (water, 20% (*v*/*v*) ethanol, or 50% (*v*/*v*) propylene glycol) at final concentrations ranging from 0.05% to 100% (*v*/*v*) was mixed with 135 μL of the ABTS working solution. As controls, 15 μL of each solvent was mixed with 135 μL of the ABTS working solution. L-ascorbic acid was used as the positive control. After 15 min of incubation in the dark at room temperature, the absorbance was measured at λ = 405 nm using a FilterMax F5 microplate reader (Molecular Devices, San Jose, CA, USA). The absorbance values were corrected by subtracting the absorbance of the corresponding sample blank (without ABTS) to account for the intrinsic color of the extracts. The percentage of ABTS•^+^ scavenging activity was calculated according to the following formula:

(5) $$ABTS\;Scavenging\;Activity\left[\%\right]=\left(1-\;\frac{Abs_{S}}{Abs_{S}}\;\;\right)\times 100\%$$

where Abs_S is the absorbance of the sample and Abs_C is the absorbance of the corresponding solvent control. The half-maximal effective concentration (EC_50_) was calculated from the dose–response curves using GraphPad Prism, version 8.4.3 for Windows (GraphPad Software, San Diego, CA, USA). All measurements were performed in triplicate.

### 3.7. Tyrosinase Inhibitory Assay

The inhibitory effect of *I. paraguariensis* and *C. sinensis* extracts on mushroom tyrosinase activity was determined according to the protocol of Uchida et al. [49] with some modifications. Briefly, 20 μL of mushroom tyrosinase solution (500 U/mL in 100 mM phosphate buffer, pH 6.8) was mixed with 120 μL of 100 mM phosphate buffer (pH 6.8) and 20 μL of the analyzed extract at final concentrations of 10%, 5%, 2.5% and 1%. Kojic acid in corresponding concentrations was used as the positive control. After 10 min of incubation at room temperature (RT), 40 μL of L-DOPA (8 mM in phosphate buffer) was added to each well. Following 15 min of incubation at RT, the absorbance was measured at λ = 450 nm using a FilterMax F5 microplate reader (Molecular Devices, San Jose, CA, USA). For each analyzed extract, the corresponding solvent was used as the control (water for the aqueous extract, 20% (*v*/*v*) ethanol for the ethanolic extract, and 50% (*v*/*v*) propylene glycol for the PG extract). The absorbance of the control sample was set as 100% tyrosinase activity, and the residual enzyme activity in the presence of the extracts was calculated according to the following formula:

(6) $$Tyrosinase\;activity\;\left[\%\right]=\;\frac{Abs_{S}}{Abs_{C}}\;\times 100\%$$

where Abs_S is the absorbance of the sample (with extract) and Abs_C is the absorbance of the corresponding solvent control (without extract). All measurements were performed in triplicate.

### 3.8. In Vitro Sun Protection Factor (SPF)

In vitro SPF of matcha and yerba mate extracts at 1%, 2.5%, and 5% (*v*/*v*) was estimated based on the absorbance values measured at 5 nm intervals in the range of λ = 290–320 nm using a DR6000 UV-Vis spectrophotometer (Hach-Lange, Wrocław, Poland), the EE(λ) × I(λ) values established by Sayre et al. [50], and the Mansur equation [25]:

(7) $$Sun\;Protection\;factor\;\left(SPF\right)=CF\;\times \;\sum_{290}^{320}EE\left(\lambda \right)\times I(\lambda )\;\times Abs\left(\lambda \right)$$

where CF is the correction factor (CF = 10), EE(λ) is the erythemal effect spectrum, I(λ) is the solar intensity spectrum, and Abs(λ) is the absorbance of the extract at wavelength λ. All measurements were performed in triplicate.

### 3.9. Critical Wavelength (λC) and UVB/UVA Ratio Calculations

To assess the spectral breadth of UV absorption matcha and yerba mate extracts (1%, 2.5%, and 5% *v*/*v* in the corresponding solvent) were scanned continuously from 200 to 400 nm at 1 nm intervals using a DR6000 UV-Vis spectrophotometer (Hach-Lange, Wrocław, Poland). The critical wavelength (λc), defined as the wavelength at which the area under the absorbance curve integrated from 290 nm reaches 90% of the total area integrated between 290 and 400 nm, was calculated according to the method of Diffey [51]:

(8) $$\int_{290}^{\lambda_{c}}A\left(\lambda \right)\;d\lambda \;=\;0.9\;\int_{290}^{400}A\left(\lambda \right)\;d\lambda$$

where A(λ) is the absorbance of the extract at wavelength λ.

Extracts with λc ≥ 370 nm are conventionally classified as providing broad-spectrum (UVB/UVA) protection [51]. As an additional descriptor of spectral balance, the ratio of the area under the absorbance curve in the UVA range (320–400 nm) to that in the UVB range (290–320 nm) was calculated for each extract.

### 3.10. In Vitro Cytotoxicity

The cytotoxic effect of the analyzed extracts was determined using the Neutral Red Uptake (NRU) assay [52]. A375 human melanoma cells were maintained in high-glucose DMEM supplemented with 10% FBS at 37 °C in a humidified atmosphere of 5% CO_2_. *Ilex paraguariensis* and *Camellia sinensis* extracts were diluted with the appropriate culture medium to final concentrations ranging from 0.625% to 10% (*v*/*v*). The cells were seeded onto 96-well plates at a density of 3000 cells/well and maintained overnight in complete growth medium. Next, the cells were treated with 100 μL of the diluted extracts or the corresponding solvent controls (water, 20% (*v*/*v*) ethanol, or 50% *(v*/*v*) propylene glycol). After 48 h of incubation, the cells were exposed for 3 h to culture medium containing Neutral Red at a final concentration of 33 μg/mL, prepared by 1:100 (*v*/*v*) dilution of the Neutral Red stock solution (3.3 g/L in DPBS). The wells were then rinsed with DPBS and lysed using an acidified ethanol solution (50% (*v*/*v*) ethanol with 1% (*v*/*v*) acetic acid) for 10 min on a microplate shaker at room temperature. The absorbance was measured at λ = 540 nm using a FilterMax F5 microplate reader (Molecular Devices, San Jose, CA, USA). The percentage of cell viability was calculated relative to the mean absorbance of the corresponding solvent controls (set as 100% viability). Experiments were performed in three independent biological replicates, with five technical replicates (wells) per tested concentration in each experiment.

### 3.11. IC_50_ and Selectivity Index Calculation

The half-maximal inhibitory concentration (IC_50_) for each extract was determined from the dose–response data (0.625–10% *v*/*v*) by linear interpolation on the log-transformed concentration scale, between the two concentrations bracketing 50% cell viability. Where cell viability did not fall below 50% within the tested concentration range, IC_50_ was reported as greater than the highest tested concentration (IC_50_ > 10%). The Selectivity Index (SI) was calculated as the ratio of the IC_50_ against normal BJ fibroblasts to the IC_50_ against A375 melanoma cells:

(9) $$Selectivity\;Index\;\left(SI\right)=\;\frac{{IC}_{50}\;(BJ\;fibroblasts)}{{IC}_{50}\;(A375\;melanoma)}$$

SI values > 1 indicate preferential cytotoxicity towards melanoma cells relative to normal fibroblasts. Where the IC_50_ for BJ fibroblasts exceeded the highest tested concentration, SI was reported as a lower-bound estimate.

### 3.12. HPLC-ESI-QTOF-MS/MS Profiling

The phytochemical profiles of all obtained extracts from *Ilex paraguariensis* (Yerba Maté) and *Camellia sinensis* (Matcha) were recorded using HPLC-ESI-QTOF-MS/MS (Agilent Technologies, Santa Clara, CA, USA). The chromatographic system (1200 Series), consisting of a binary pump, a degasser, an autosampler, a column thermostat, and a DAD detector, was coupled with a Q-TOF mass spectrometer (G6530B, Agilent Technologies) operating in both positive and negative ionisation modes. Compound identification was performed using MassHunter software (version B.12.00, Agilent Technologies), based on high-accuracy mass measurements and built-in molecular formula generators (mass error tolerance ≤15 ppm), supported by published literature and publicly available mass spectrometric databases.

Chromatographic separation was performed on a ReproSil-Pur 120 C18-AQ column (Dr. Maisch GmbH, Ammerbuch, Germany; 150 mm × 4.6 mm, 5 µm) using a binary gradient of 0.1% aqueous formic acid (solvent A) and acetonitrile with 0.1% formic acid (solvent B) at a flow rate of 0.2 mL/min and column temperature of 20 °C, followed by a 5 min post-run equilibration. The gradient program was as follows: 0 min: 10% B; 10 min: 20% B; 15 min: 40% B; 17 min: 95% B; 18 min: 95% B; 19–30 min: 10% B. The mass spectrometer was operated under the following conditions: *m*/*z* range 100–1700 Da, gas and sheath gas temperatures of 275 °C and 325 °C, gas flow 12 L/min, nebulizer pressure 35 psig, capillary voltage 3000 V, fragmentor voltage 110 V, skimmer voltage 65 V, and collision energy 10 V. All extracts were analyzed in triplicate in both ionization modes.

### 3.13. Statistical and Chemometric Analysis

#### 3.13.1. Statistical Analysis

All quantitative results were expressed as mean ± standard deviation (SD). Statistical analysis was performed for SPF, DPPH radical-scavenging activity, tyrosinase inhibition, A375 melanoma cytotoxicity and fibroblast cytotoxicity. SPF, DPPH radical-scavenging activity and tyrosinase-inhibition assays were performed in triplicate (*n* = 3), whereas the A375 melanoma and fibroblast viability assays were performed in three independent biological replicates, with five technical replicates (wells) per tested concentration in each experiment.

The effect of extraction solvent was evaluated separately within each plant matrix using one-way ANOVA followed by Tukey’s honestly significant difference (HSD) post hoc test. In parallel, two-way ANOVA was used to assess the effects of plant material, extraction solvent and their interaction. Normality within groups was evaluated using the Shapiro–Wilk test, and homogeneity of variance was assessed using Levene’s test. Differences were considered statistically significant at *p* < 0.05. Groups sharing the same letter were considered not significantly different according to Tukey’s HSD test. Due to one missing replicate for the propylene glycol extracts in the DPPH assay, these groups were analyzed with *n* = 2 and interpreted cautiously.

#### 3.13.2. Chemometric Dataset and Preprocessing

Chemometric analysis was performed using a phytochemical dataset comprising six standardized extracts and thirteen variables. The analyzed extracts were Matcha H_2_O, Matcha EtOH, Matcha PG, Yerba mate H_2_O, Yerba mate EtOH and Yerba mate PG. The variables included eleven selected LC–MS phytochemical markers together with total polyphenol and total flavonoid contents. Before multivariate analysis, the data were autoscaled column-wise using z-score transformation, calculated as z = (x − mean)/SD, where SD was the sample standard deviation. Autoscaling was applied to remove scale-dependent effects and to allow variables with different absolute response ranges to contribute comparably to the analysis.

#### 3.13.3. Hierarchical Clustering and Czekanowski Diagram

Hierarchical clustering analysis (HCA) was used to compare the overall phytochemical profiles of the six extracts. Pairwise Euclidean distances were calculated from the autoscaled data matrix, and agglomerative hierarchical clustering was performed using Ward’s linkage method, which aims to minimize within-cluster variance during cluster fusion [53,54]. The dendrogram was generated in Python 3.12.3 using custom scripts based on NumPy 2.4.4, pandas 3.0.2, SciPy 1.17.1 and Matplotlib 3.10.8. Euclidean distances were calculated using scipy.spatial.distance.pdist, transformed into a square distance matrix using squareform, and clustered using scipy.cluster.hierarchy.linkage. The dendrogram was visualized using scipy.cluster.hierarchy.dendrogram and exported as high-resolution graphical output.

An ordered Czekanowski diagram was constructed to visualize similarity relationships between phytochemical variables. For this purpose, the phytochemical data matrix was arranged so that the thirteen variables were treated as objects and the extract profiles were used as descriptors. Pairwise Euclidean distances between variables were calculated after autoscaling and then transformed into symbolic similarity classes using predefined distance thresholds. Distances ≤ 0.5 were marked with “#”, distances > 0.5–1.0 with “+”, distances > 1.0–1.5 with “−”, and distances > 1.5 were left blank. Diagonal elements were marked with “#”. The symbolic matrix was ordered according to hierarchical clustering to improve visualization of similarity blocks. The Czekanowski diagram was generated using a custom Python 3.12.3 script with NumPy 2.4.4, pandas 3.0.2, SciPy 1.17.1 and Matplotlib 3.10.8; symbolic classes were visualized using a discrete grayscale colormap.

#### 3.13.4. PCA-Based Regression Modelling and LOOCV

Principal component analysis (PCA) followed by VARIMAX rotation was used to reduce the dimensionality of the phytochemical dataset and to obtain latent factors F1–F3 [3]. PCA-derived factor loadings were subsequently used as explanatory variables in regression modelling of the biological and cosmetic endpoints, including tyrosinase inhibition, in vitro SPF, fibroblast cytotoxicity, melanoma cytotoxicity and DPPH radical-scavenging activity. Only models showing satisfactory fitting and cross-validation parameters were retained for detailed interpretation; therefore, final interpretable models were obtained for tyrosinase inhibition, SPF and fibroblast cytotoxicity. Model quality was evaluated using the model standard deviation, correlation coefficient (R), determination coefficient (D/R^2^) and F-test value.

Predictive stability was assessed by leave-one-out cross-validation (LOOCV), a validation approach in which each object is omitted once, the model is fitted to the remaining *n* − 1 objects, and the omitted object is predicted by a model that was not built using that observation [55]. In each LOOCV round, autoscaling was performed using only the training subset in order to avoid information leakage from the validation object into the model. Predictive performance was evaluated using the predicted residual sum of squares (PRESS), cross-validated coefficient of determination (Q^2^_LOO), standard deviation error of prediction (SDEP_LOO), root mean square error of cross-validation (RMSECV_LOO) and mean absolute error (MAE_LOO). PRESS represents the sum of squared differences between experimental and LOOCV-predicted values. Q^2^_LOO is the predictive analogue of R^2^ and was calculated as 1 − PRESS/Σ(y_i_ − ȳ)^2^. SDEP_LOO and RMSECV_LOO quantify the average prediction error in the units of the response variable, whereas MAE_LOO represents the mean absolute prediction error and is less sensitive to individual large residuals than squared-error metrics.

## Figures and Tables

**Table 1:** Comparison of total polyphenolic content (TPC), total flavonoid content (TFC) and antioxidant potential of matcha and yerba mate extracts; data are presented as mean ± SD (*n* = 3); values within the same row followed by the same superscript letter are not significantly different (*p* > 0.05). * EC_50_ value below the lowest concentration tested (0.02% *v*/*v*); therefore, SD and statistical comparison could not be determined for this sample.

**Matcha (*C. sinensis*) Extracts**				
Green solvent	H_2_O	20% EtOH	50% PG	Vitamin C
TPC (mg GAE/mL extract)	1.37 ± 0.04 ^a^	1.28 ± 0.07 ^b^	1.35 ± 0.03 ^c^	-
TFC (mg QE/mL extract)	0.16 ± 0.00 ^d^	0.15 ± 0.01 ^d^	0.31 ± 0.01 ^e^	-
EC_50_ (DPPH, in %)	0.07 ± 0.01 ^f^	0.04 ± 0.01 ^f^	0.31 ± 0.10 ^g^	<0.02 *
EC_50_ (ABTS, in %)	0.11 ± 0.01 ^h^	0.04 ± 0.00 ^i^	<0.02 *	<0.02 *
**Yerba mate (*I. paraguariensis*) extracts**				
Green solvent	H_2_O	20% EtOH	50% PG	Vitamin C
TPC (mg GAE/mL)	1.43 ± 0.07 ^a^	1.54 ± 0.02 ^b^	1.54 ± 0.01 ^b^	-
TFC (mg QE/mL)	0.84 ± 0.03 ^d^	0.59 ± 0.05 ^e^	0.95 ± 0.04 ^f^	-
EC_50_ (DPPH, in %)	0.07 ± 0.01 ^g^	0.07 ± 0.01 ^g^	0.67 ± 0.05 ^h^	<0.02 *
EC_50_ (ABTS, in %)	0.07 ± 0.01 ^i^	0.18 ± 0.02 ^j^	0.06 ± 0.01 ^i^	<0.02 *

**Table 2:** Comparison of the in vitro sun protection factor (SPF) of matcha and yerba mate extracts; values in the table indicate mean ± SD; *n* = 3.

**Matcha (*C. sinensis*) Extracts**			
Green solvent	H_2_O	20% EtOH	50% PG
5%	11.74 ± 0.23	11.50 ± 0.05	18.54 ± 0.53
2.5%	5.77 ± 0.31	4.96 ± 0.01	8.60 ± 0.08
1%	1.97 ± 0.01	2.52 ± 1.64	2.91 ± 0.07
**Yerba mate (*I. paraguariensis*) extracts**			
Green solvent	H_2_O	20% EtOH	50% PG
5%	27.87 ± 0.19	19.53 ± 2.87	28.65 ± 0.11
2.5%	21.91 ± 0.05	27.55 ± 0.99	26.19 ± 0.21
1%	8.81 ± 3.01	18.12 ± 1.53	10.40 ± 1.67

**Table 4:** Compounds’ assignment based on the HR mass measurements and comparison of relative abundance of phenolic compounds in different matcha extracts (*Camellia sinensis*) based on LC–MS peak areas. Relative abundance was expressed as a LC-MS peak area values and semi-quantitatively as: Tr (trace, <10^6^), + (low, 10^6^–10^7^), ++ (moderate, 10^7^–10^8^), and +++ (high, >10^8^); nd—not detected; (Rt-retention time, *m*/*z* calc—theoretical ion mass, *m*/*z* exp—experimental mass, DBE—number of double bonds and rings, error—error of measurement, MSI level—Metabolomics Standards Initiative level: MSI level 2a—putatively annotated compounds based on accurate mass and MS/MS inhouse library/literature matching, with an authentic standard in the inhouse library. MSI level 2b—putatively characterized compound classes, based on the MS/MS fragmentation, inhouse and commonly available mass banks).

No	Ion Mode	RT [min]	Formula	*m*/*z* calc	*m*/*z* exp	Error	DBE	MS/MS Fragments	Compound	Class	H_2_O	20% EtOH	50% PG	MSI Level
1	+	2.18	C_7_H_14_N_2_O_3_	175.1077	175.107	−4.13	2	158.0818 129.1030	Theanine	Amino acids	++	++	++	2b
2	+	2.61	C_14_H_16_O_9_	345.0816	345.0793	−6.75	7	153.0182 125.0225	Theogallin	Polyphenols	+	+	+	2b
3	+	2.78	C_7_H_8_N_4_O_2_	181.072	181.072-	4.99	6	165.0539 147.0468 136.0765	Theobromine	Alkaloids	+	+	+	2a
4	+	3.11	C_14_H_20_O_8_	301.1282	301.1272	−3.26	5	242.0766 142.0141	Tyrosol-*O*-Glucoside	Phenolic compounds	Tr	Tr	nd	3
5	-	12.59	C_15_H_14_O_7_	305.0667	305.0660	−2.21	9	287.0506 219.0662 179.0329 137.0242 125.0236	Gallocatechin	Catechins	+	++	++	2a
6	-	12.80	C_22_H_18_O_11_	457.0776	457.0768	−1.82	14	305.0590 269.0382 169.0091	Epigallocatechin Gallate	Catechins	++	+++	+++	2a
7	+	13.07	C_21_H_20_O_10_	433.1188	433.1149	4.57	12	415.1036 313.0743 283.0637 271.0623	Vitexin	Flavonoids	+	+	+	2b
8	+	13.39	C_21_H_20_O_12_	465.1028	465.1020	−1.62	12	303.0513 127.0395	Quercetin Hexoside	Flavonoids	+	+	+	2b
9	-	13.42	C_16_H_18_O_9_	353.087	353.0882	1.11	8	191.0565 179.0349 135.0454	3-*O*-Caffeoyl-quinic Acid	Phenolic acids	++	++	++	2a
10	+	13.7	C_21_H_20_O_12_	465.1028	465.1017	−2.27	12	303.0534 145.0491 127.0402	Isoquercitrin	Flavonoids	+	+	+	2a
11	-	14.6	C_15_H_14_O_7_	305.0667	305.0671	1.38	9	287.0512 219.0655 179.0339 137.0240	Gallocatechin	Catechins	+	++	++	2a
12	+	14.78	C_8_H_10_N_4_O_2_	195.0877	195.0867	−4.91	6	138.0656 110.0710	Caffeine	Alkaloids	+++	+++	+++	2a
13	-	15.27	C_16_H_18_O_9_	353.0878	353.0888	2.81	8	191.0550 173.0439 135.0439	5-*O*-Caffeoyl-quinic Acid	Phenolic acids	++	++	++	2a
14	-	15.52	C_15_H_14_O_6_	289.0718	289.0725	2.55	9	245.0802 203.0699 179.0340 137.0232	Epicatechin	Catechins	+	++	++	2a
15	-	16.04	C_21_H_20_O_11_	447.0933	447.0898	−7.78	12	357.0513 327.0441 285.0365	Kaempferol-*O*-Galactoside	Flavonoids	+	++	+	2a
16	-	16.95	C_30_H_28_O_12_	579.1497	579.1516	1.38	17	289.0717 245.0817 203.0715	Gambiriin A1	Tannins	+	+	+	2b
17	-	18.64	C_21_H_20_O_11_	447.0933	447.0933	0.33	12	327.0536 284.0319 255.0283	Kaempferol hexoside	Flavonoids	+	+	+	2b
18	-	19.29	C_22_H_18_O_10_	441.0827	441.0828	0.18	14	289.0715 169.0140 125.0239	Epicatechin Gallate	Catechins	+	++	++	2a

**Table 5:** Compounds’ assignment based on the HR mass measurements and comparison of relative abundance of phenolic compounds in different yerba mate (*Ilex paraguariensis)* extracts based on LC–MS peak areas. Relative abundance was expressed as a LC-MS peak area values and semi-quantitatively as: Tr (trace, <10^6^), + (low, 10^6^–10^7^), ++ (moderate,10^7^–10^8^), and +++ (high, >10^8^); nd—not detected; (Rt-retention time, *m*/*z* calc—theoretical ion mass, *m*/*z* exp—experimental mass, DBE—number of double bonds and rings, error—error of measurement, MSI level—Metabolomics Standards Initiative level: MSI level 2a—putatively annotated compounds based on accurate mass and MS/MS inhouse library/literature matching, with an authentic standard in the inhouse library. MSI level 2b—putatively characterized compound classes, based on the MS/MS fragmentation, inhouse and commonly available mass banks).

No	Ion	RT [min]	Formula	*m*/*z* calc	*m*/*z* exp	Error	DBE	MS/MS Fragments	Compound	Class	H_2_O	20% EtOH	50% PG	MSI Level
1	+	2.1	C_7_H_14_N_2_O_3_	175.1077	175.1075	−1.256	2	158.0822 129.1032 84.0453	Theanine	Amino acids	Tr	+	+	2b
2	+	2.77	C_7_H_8_N_4_O_2_	181.072	181.0714	−3.34	6	163.0619 138.0671 110.0724	Theobromine	Alkaloids	++	++	+	2b
3	+	3.10	C_14_H_20_O_8_	301.1282	301.1279	−0.93	5	242.0766 142.0141	Tyrosol-*O*-Glucoside	Phenolic compounds	+	Tr	Tr	3
4	+	10.39	C_14_H_16_O_9_	345.0816	345.079	−7.62	7	153.0179 125.0233	Theogallin	Polyphenols	Tr	Tr	nd	2b
5	+	11.52	C_33_H_40_O_21_	773.2135	773.2161	1.96	14	611.1620 465.1066 303.0526	-Quercetin-rhamnodiglucoside isomer 1	Flavonoids	+	Tr	+	2b
6	+	11.90	C_33_H_40_O_21_	773.2135 773.2135	773.2163	2.22	14	611.1686 465.1076 303.0519	Quercetin-rhamnodiglucoside isomer 2	Flavonoids	nd	Tr	++	2b
7	-	13.3	C_16_H_18_O_9_	353.0878	353.0890	3.37	8	191.0567 179.0357 135.0456	3-*O*-Caffeoylquinic acid	Phenolic acids	+++	+++	++	2a
8	-	14.68	C_15_H_14_O_7_	305.0667	305.0654	−4.17	9	219.0654 179.0336 167.0342 137.0238 125.0235	Gallocatechin	Catechins	Tr	+	++	2a
9	+	15.3	C_8_H_10_N_4_O_2_	195.0877	195.0878	0.76	6	138.0660 110.0714	Caffeine	Alkaloids	+++	+++	+++	2a
10	-	15.35	C_16_H_18_O_9_	353.0878	353.0885	1.96	8	191.0554 173.0441 161.0234	5-*O*-Caffeoylquinic acid	Phenolic acids	+++	+++	++	2a
11	-	16.95	C_15_H_14_O_6_	289.0718	289.0729	3.92	9	245.0804 203.0694 137.0236	Epicatechin	Catechins	Tr	+	++	2a
12	-	17.46	C_16_H_18_O_8_	337.0929	337.0939	2.98	8	191.0563 173.0455 137.0231	Coumaroylquinic Acid	Phenolic acids	+	+	+	2b
13	-	17.62	C_17_H_20_O_9_	367.1035	367.1032	−0.69	8	193.0508 173.0452 134.0369	Feruloylquinic Acid	Phenolic acids	Tr	+	+	2b
14	-	17.7	C_33_H_40_O_20_	755.2040	755.2032	−1.08	14	285.0347	Kaempferol rhamnodiglucoside isomer 1	Flavonoids	Tr	Tr	Tr	2b
15	-	18.2	C_33_H_40_O_20_	755.204	755.2023	−2.27	14	285.0339	Kaempferol hexoside isomer 2	Flavonoids	Tr	Tr	Tr	2b
16	+	18.14	C_27_H_30_O_16_	611.1607	611.1616	1.54	13	303.0518 129.0560	Rutin	Flavonoids	+	++	++	2a
17	-	18.6	C_34_H_30_O_15_	677.1512	677.1524	1.78	20	-	Tricaffeoylquinic Acid	Phenolic acids	Tr	+	Tr	2a
18	-	19.3	C_22_H_18_O_10_	441.0827	441.0828	0.18	14	331.0449 289.0715 169.0140	Epicatechin Gallate	Catechins	Tr	+	+	2a
19	-	19.55	C_25_H_24_O_12_	515.1195	515.1192	−0.58	14	353.0879 191.0568 179.0352 135.0454	3,5-Dicaffeoylquinic Acid	Phenolic acids	++	++	+	2a
20	+	19.59	C_27_H_30_O_16_	595.1657	595.1661	0.59	13	449.1103 287.0573 129.0552	Kaempferol-*O*-Galactoside	Flavonoids	Tr	++	+	2b
21	-	21.02	C_27_H_28_O_13_	559.1457	559.1448	−1.63	14	397.1018 301.0286 173.0401	Caffeoyl-feruloylquinic acid	Phenolic acids	Tr	Tr	Tr	2a

**Table 6:** Model-derived associations between phytochemical patterns and selected cosmetic endpoints. Higher tyrosinase inhibition and SPF are desirable responses, whereas higher fibroblast cytotoxicity is undesirable. Associations were inferred from PCA-regression coefficients and should not be interpreted as proof of causality for individual compounds.

Endpoint	Phytochemical Pattern Associated with a Higher Response	Phytochemical Pattern Associated with a Lower Response	Formulation-Oriented Interpretation
Tyrosinase inhibition	Theanine; theogallin; tyrosol-*O*-glucoside	Total polyphenols; total flavonoids; kaempferol-*O*-galactoside; caffeoylquinic acid isomers	The model supports a Matcha-like marker pattern for future brightening-oriented optimization.
In vitro SPF	Total flavonoids; tyrosol-*O*-glucoside; caffeoylquinic acid isomers	Theanine; theogallin	The relationship is exploratory; these markers can guide screening for UV-absorbing formulations.
Fibroblast cytotoxicity	Theobromine; theanine; theogallin; caffeoylquinic acid isomers	Caffeine; epicatechin; epicatechin gallate; gallocatechin	Marker patterns associated with lower cytotoxicity should be prioritized during safety-oriented formulation development.

## Data Availability

Data is contained within the article and Supplementary Materials.

## Supplementary Materials

The following supporting information can be downloaded at: https://www.mdpi.com/article/10.3390/molecules31162742/s1, Figure S1. Representative HPLC-ESI-QTOF-MS/MS chromatograms (negative ionization mode) of Matcha (*Camellia sinensis*) extracts obtained using three different extraction solvents: (A) H_2_O, (B) 20% EtOH, and (C) 50% PG (propylene glycol); Figure S2. Representative HPLC-ESI-QTOF-MS/MS chromatograms of Yerba Maté (*Ilex paraguariensis*) extracts obtained using three different extraction solvents in negative (A–C) and positive (D–F) ionization mode: (A,D) H_2_O, (B,E) 20% EtOH, and (C,F) 50% PG (propylene glycol); Figure S3. Representative HPLC-ESI-QTOF-MS/MS chromatograms of Yerba Maté (*Ilex paraguariensis*) extracts obtained using three different extraction solvents in positive ionization mode: (A) H_2_O, (B) 20% EtOH, and (C) 50% PG (propylene glycol); Figure S4. Representative HPLC-ESI-QTOF-MS/MS chromatograms (positive ionization mode) of Matcha (*Camellia sinensis*) extracts obtained using three different extraction solvents: (A) H_2_O, (B) 20% EtOH, and (C) 50% PG (propylene glycol). Figure S5. UV absorption spectra (200–400 nm) of Matcha (*Camellia sinensis*) extracts obtained using three different extraction solvents: (A) H_2_O, (B) 20% EtOH, and (C) 50% PG (propylene glycol) at three concentrations (1, 2.5 and 5%; *v*/*v*). Each curve represents the mean absorbance of three independent measurements. Figure S6. UV absorption spectra (200–400 nm) of Yerba Maté (*Ilex paraguariensis*) extracts obtained using three different extraction solvents: (A) H_2_O, (B) 20% EtOH, and (C) 50% PG (propylene glycol) at three concentrations (1, 2.5 and 5%; *v*/*v*). Each curve represents the mean absorbance of three independent measurements. Table S1. The MS/MS spectra of all tentatively identified components; Table S2. Descriptive statistics and Tukey HSD compact letter display within each plant material; Table S3. Two-way ANOVA summary for the effects of plant material, extraction solvent and their interaction; Table S4. PCA-derived loadings of phytochemical variables on latent factors F1–F3 used in bioactivity regression models; Table S5. Performance parameters of chemometric regression models and leave-one-out cross-validation. Table S6. Critical wavelength (λc) and UVA/UVB absorbance ratio of matcha (*Camellia sinensis*) and yerba mate (*Ilex paraguariensis*) extracts prepared in water, 20% (*v*/*v*) ethanol and 50% (*v*/*v*) propylene glycol (PG), calculated from UV absorption spectra recorded at 1 nm intervals between 200 and 400 nm.

## Abbreviations

The following abbreviations are used in this manuscript:

ABTS	2,2′-azino-bis(3-ethylbenzothiazoline-6-sulfonic acid)
ANOVA	Analysis of variance
ASE	Accelerated solvent extraction
ATCC	American Type Culture Collection
DBE	Double bond equivalents
DMEM	Dulbecco’s Modified Eagle’s Medium
DPBS	Dulbecco’s phosphate-buffered saline
DPPH	2,2-diphenyl-1-picrylhydrazyl
DW	Dry weight
EC_50_	Half-maximal effective concentration
EGCG	Epigallocatechin gallate
EMEM	Eagle’s Minimum Essential Medium
EtOH	Ethanol
FBS	Fetal bovine serum
GAE	Gallic acid equivalents
HCA	Hierarchical clustering analysis
HPLC-ESI-QTOF-MS	High-performance liquid chromatography–electrospray ionization–quadrupole time-of-flight mass spectrometry
INCI	International Nomenclature of Cosmetic Ingredients
LC-MS	Liquid chromatography–mass spectrometry
L-DOPA	3,4-dihydroxy-L-phenylalanine
LOOCV	Leave-one-out cross-validation
MS/MS	Tandem mass spectrometry
NaDES	Natural deep eutectic solvents
NRU	Neutral red uptake
PCA	Principal component analysis
PG	Propylene glycol
PRESS	Predicted residual sum of squares
QE	Quercetin equivalents
ROS	Reactive oxygen species
RT	Room temperature
SD	Standard deviation
SPF	Sun protection factor
TFC	Total flavonoid content
TPC	Total phenolic content
UAE	Ultrasound-assisted extraction
UV	Ultraviolet
UVB	Ultraviolet B
λc	Critical wavelength
