# Supplementary material for: Cosmeceutical Potential of Matcha (Camellia sinensis) and Yerba Mate (Ilex paraguariensis) Extracts Obtained with Green Solvents
Source: Molecules. 2026 Aug 7;31(16):2742. doi: 10.3390/molecules31162742 (PMC13515746; doi:10.3390/molecules31162742)
Supplement: Supplementary file 1 [file molecules-31-02742-s001.zip › molecules-4452458-supplementary.pdf]

Supplementary Materials

for

Cosmeceutical Potential of Matcha (*Camellia sinensis*) and Yerba Mate (*Ilex paraguariensis*) Extracts Obtained with Green Solvents

Katarzyna Gaweł-Bęben[1*], Monika Szczepanik[1], Paweł Szczeblewski[2], Karolina Czech[1], and Wirginia Kukula-Koch[3]

Contents

- **Figure S1**. Representative HPLC-ESI-QTOF-MS/MS chromatograms (negative ionization mode) of Matcha (*Camellia sinensis*) extracts obtained using three different extraction solvents: (A) H_2O, (B) 20% EtOH, and (C) 50% PG (propylene glycol).
- **Figure S2**. Representative HPLC-ESI-QTOF-MS/MS chromatograms of Yerba Maté (*Ilex paraguariensis*) extracts obtained using three different extraction solvents in negative (A–C) and positive (D–F) ionization mode: (A,D) H_2O, (B,E) 20% EtOH, and (C,F) 50% PG (propylene glycol).
- **Figure S3**. Representative HPLC-ESI-QTOF-MS/MS chromatograms of Yerba Maté *(Ilex paraguariensis*) extracts obtained using three different extraction solvents in positive ionization mode: (A) H_2O, (B) 20% EtOH, and (C) 50% PG (propylene glycol).
- **Figure S4**. Representative HPLC-ESI-QTOF-MS/MS chromatograms (positive ionization mode) of Matcha (*Camellia sinensis*) extracts obtained using three different extraction solvents: (A) H_2O, (B) 20% EtOH, and (C) 50% PG (propylene glycol).
- **Figure S5**. UV absorption spectra (200–400 nm) of Matcha (*Camellia sinensis*) extracts obtained using three different extraction solvents: (A) H_2O, (B) 20% EtOH, and (C) 50% PG (propylene glycol) at three concentrations (1, 2.5 and 5%; *v*/*v*). Each curve represents the mean absorbance of three independent measurements.
- **Figure S6**. UV absorption spectra (200–400 nm) of Yerba Maté (*Ilex paraguariensis*) extracts obtained using three different extraction solvents: (A) H_2O, (B) 20% EtOH, and (C) 50% PG (propylene glycol) at three concentrations (1, 2.5 and 5%; *v*/*v*). Each curve represents the mean absorbance of three independent measurements.
- **Table S1**. The MS/MS spectra of all tentatively identified components.
- **Table S2**. Descriptive statistics and Tukey HSD compact letter display within each plant material.
- **Table S3**. Two-way ANOVA summary for the effects of plant material, extraction solvent and their interaction.
- **Table S4**. PCA-derived loadings of phytochemical variables on latent factors F1–F3 used in bioactivity regression models.
- **Table S5.** Performance parameters of chemometric regression models and leave-one-out cross-validation.
- **Table S6.** Critical wavelength (λc) and UVA/UVB absorbance ratio of matcha (*Camellia sinensis*) and yerba mate (Ilex paraguariensis) extracts prepared in water, 20% (*v*/*v*) ethanol and 50% (*v*/*v*) propylene glycol (PG), calculated from UV absorption spectra recorded at 1 nm intervals between 200 and 400 nm.

Figure S1. Representative HPLC-ESI-QTOF-MS/MS chromatograms (negative ionization mode) of Matcha (*Camellia sinensis*) extracts obtained using three different extraction solvents: (A) H_2O, (B) 20% EtOH, and (C) 50% PG (propylene glycol).

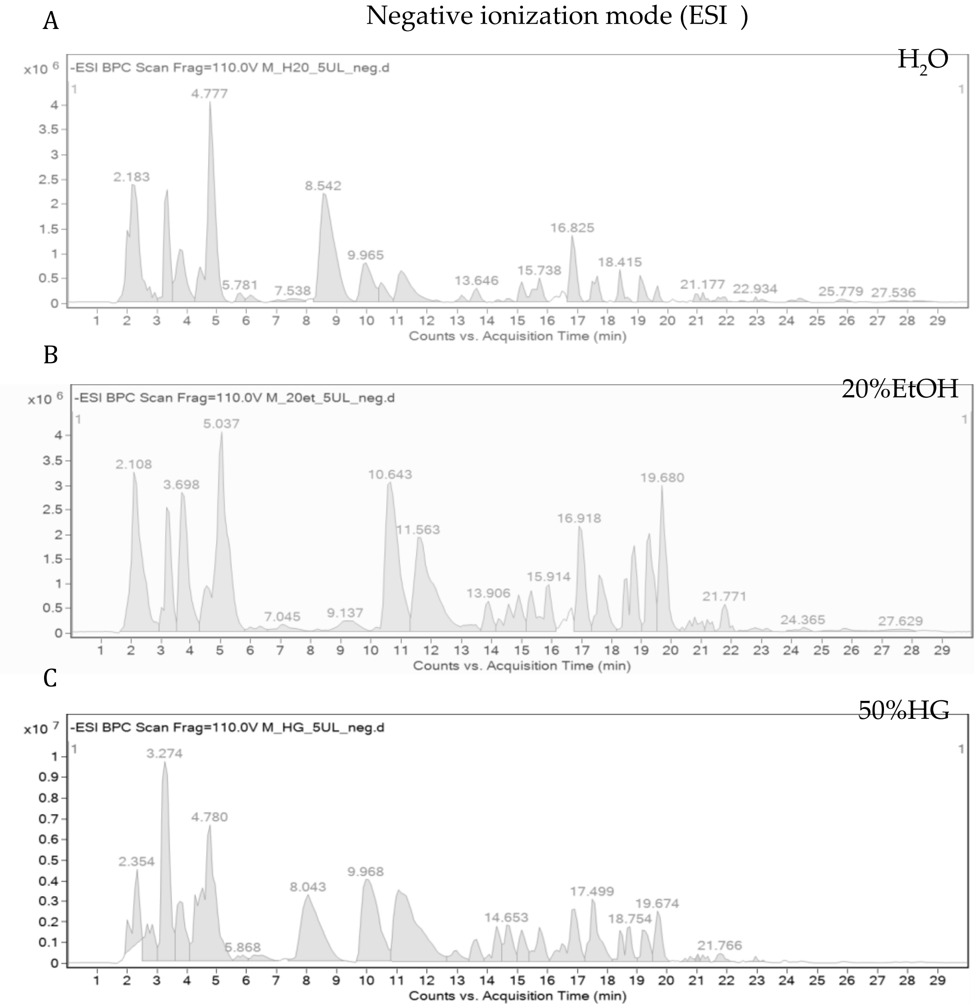

Figure S2. Representative HPLC-ESI-QTOF-MS/MS chromatograms of Yerba Maté (*Ilex paraguariensis*) extracts obtained using three different extraction solvents in negative (A–C) and positive (D–F) ionization mode: (A,D) H_2O, (B,E) 20% EtOH, and (C,F) 50% PG (propylene glycol).

Negative ionization mode (ESI $^{+}$)

A

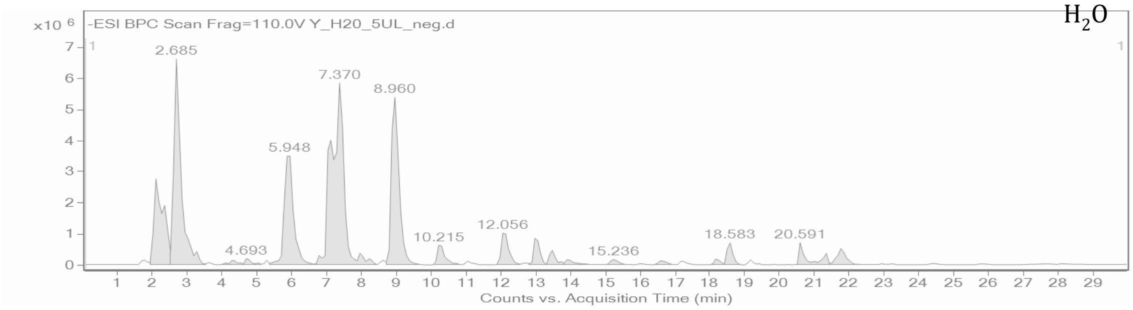

B

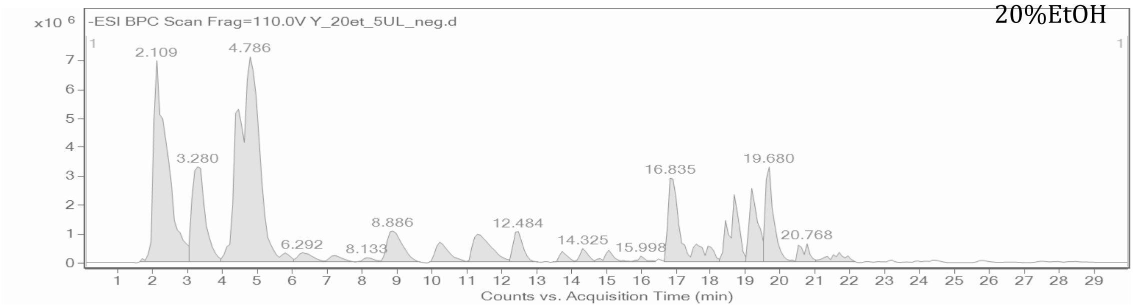

C

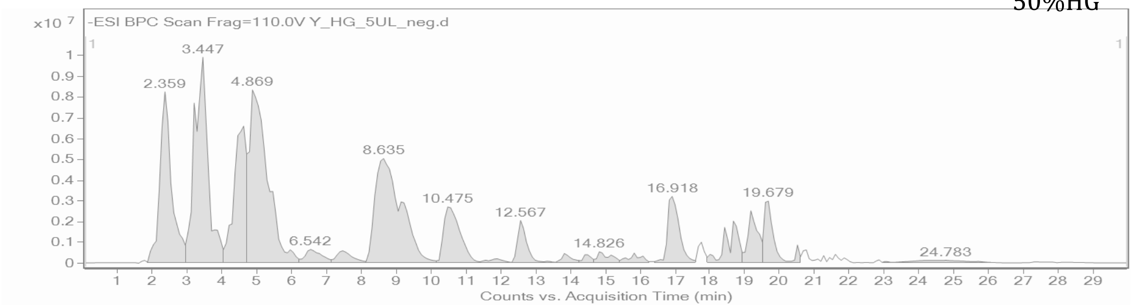

Figure S3. Representative HPLC-ESI-QTOF-MS/MS chromatograms of Yerba Maté *(Ilex paraguariensis)* extracts obtained using three different extraction solvents in positive ionization mode: (A) H_2O, (B) 20% EtOH, and (C) 50% PG (propylene glycol).

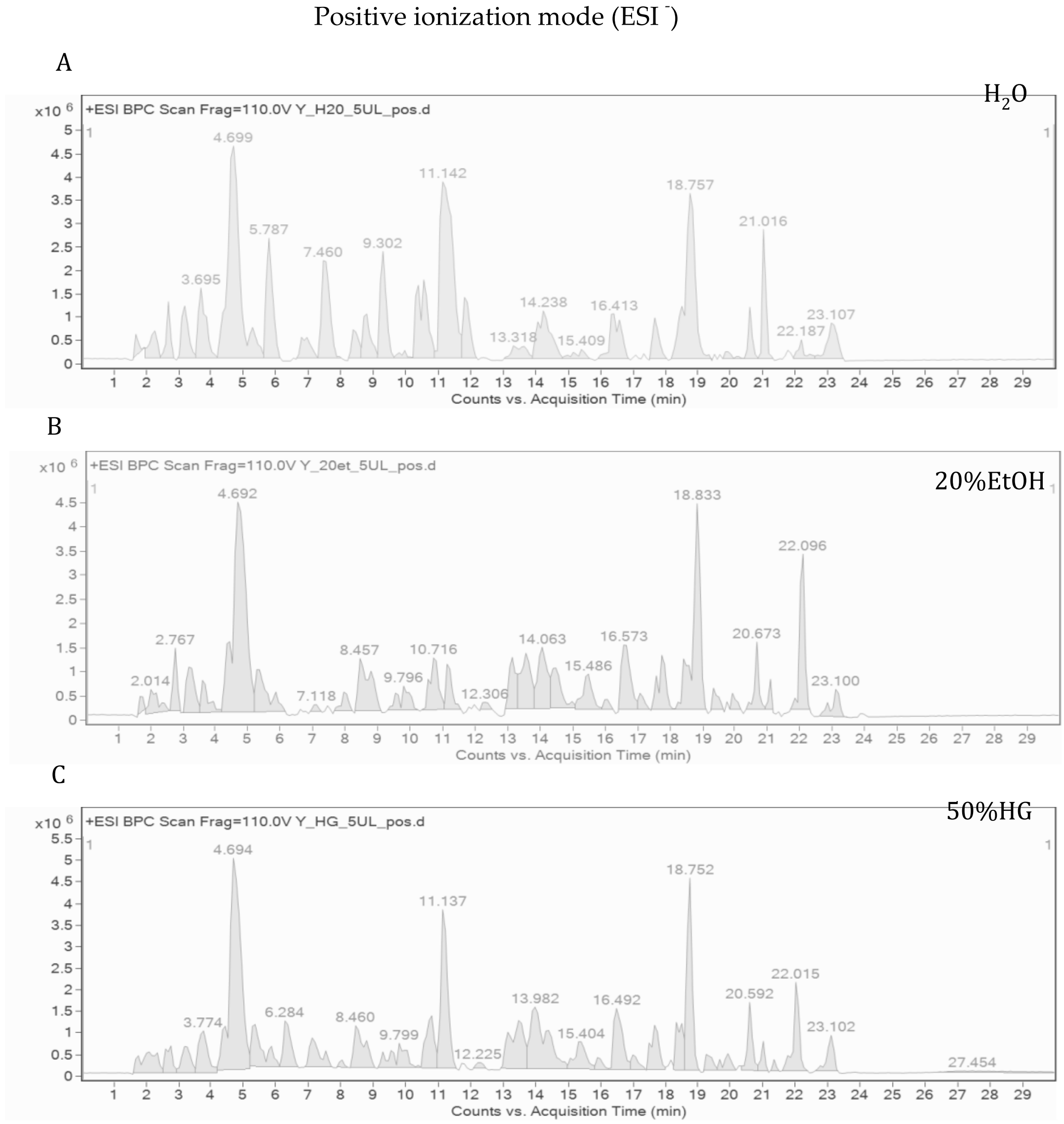

Figure S4. Representative HPLC-ESI-QTOF-MS/MS chromatograms (positive ionization mode) of Matcha (*Camellia sinensis*) extracts obtained using three different extraction solvents: (A) H_2O, (B) 20% EtOH, and (C) 50% PG (propylene glycol).

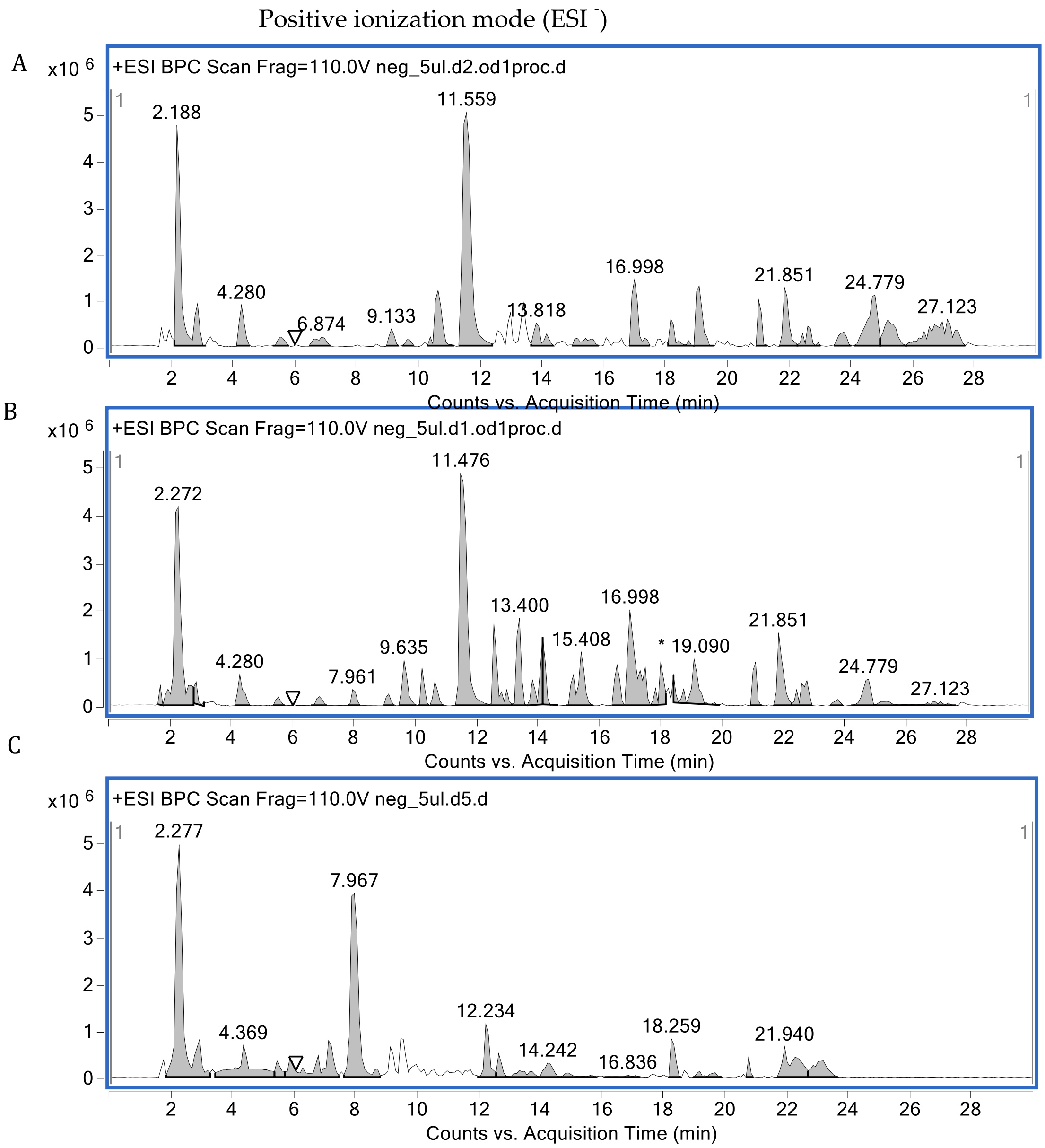

Figure S5. UV absorption spectra (200–400 nm) of Matcha (*Camellia sinensis*) extracts obtained using three different extraction solvents: (A) H_2O, (B) 20% EtOH, and (C) 50% PG (propylene glycol) at three concentrations (1, 2.5 and 5%; *v*/*v*). Each curve represents the mean absorbance of three independent measurements.

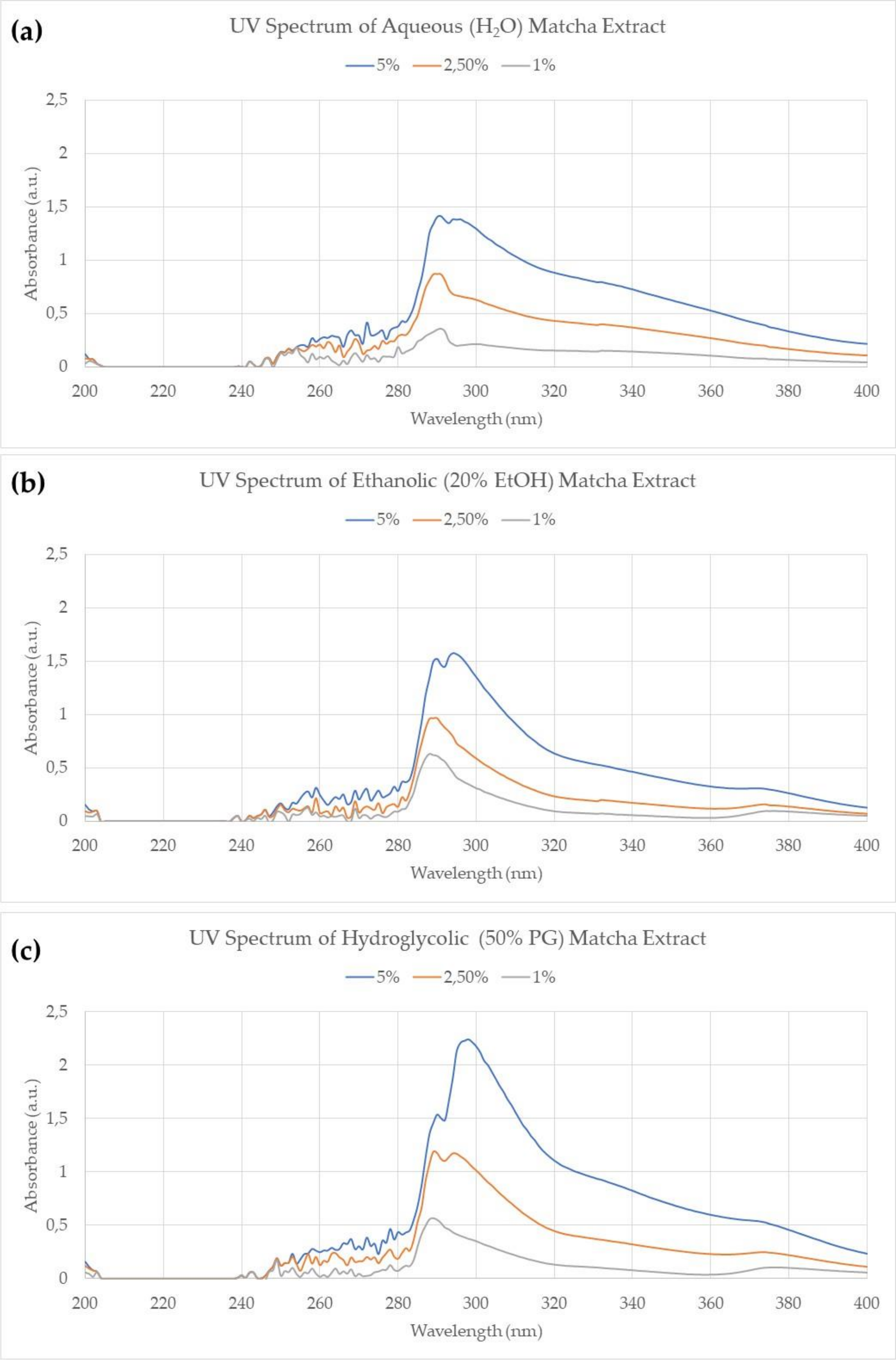

Figure S6. UV absorption spectra (200–400 nm) of Yerba Maté (*Ilex paraguariensis*) extracts obtained using three different extraction solvents: (A) H_2O, (B) 20% EtOH, and (C) 50% PG (propylene glycol) at three concentrations (1, 2.5 and 5%; *v/v*). Each curve represents the mean absorbance of three independent measurements.

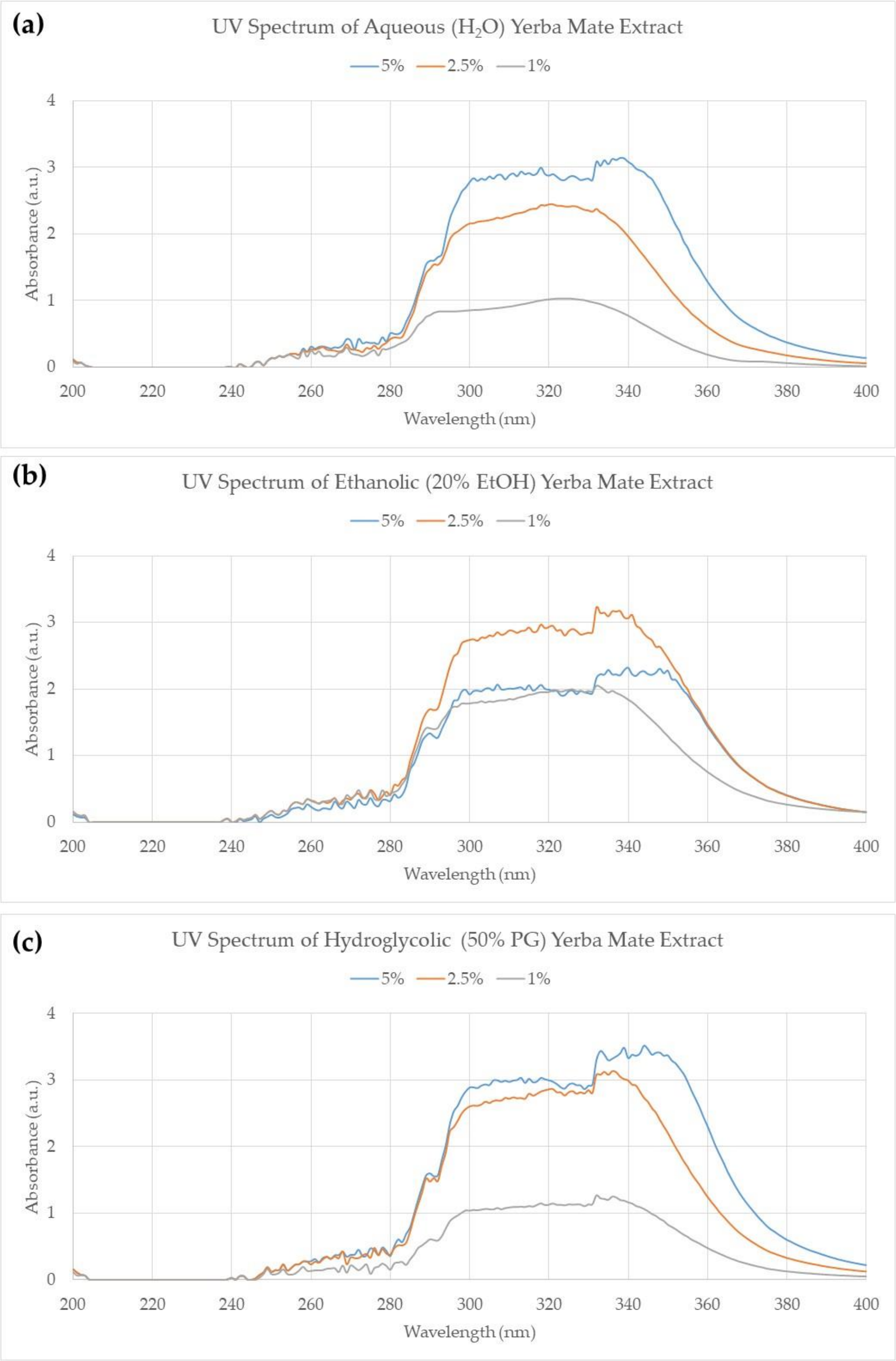

Table S1. The MS/MS spectra of all tentatively identified components.

Name	MS/MS spectrum
**3,5-Dicaffeoylquinic Acid**	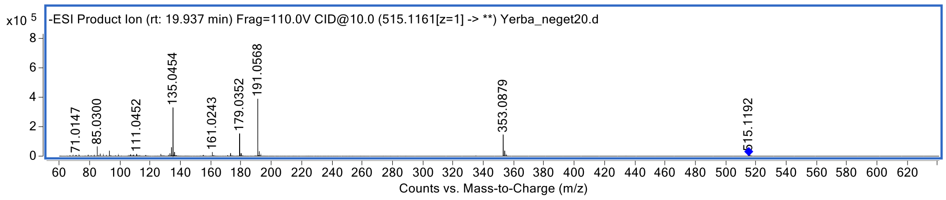
**3-*O*-Caffeoylquinic Acid**	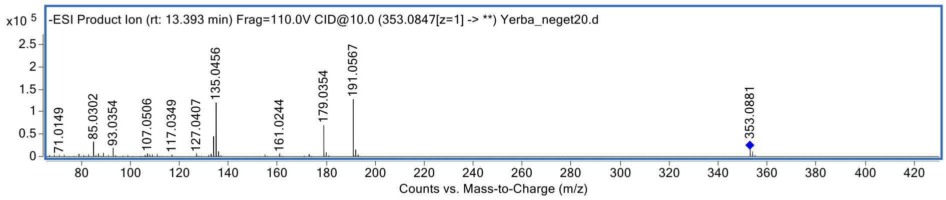
**5-*O*-Caffeoylquinic Acid**	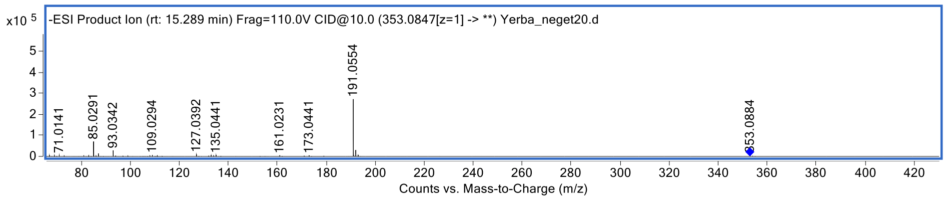
**Caffeine**	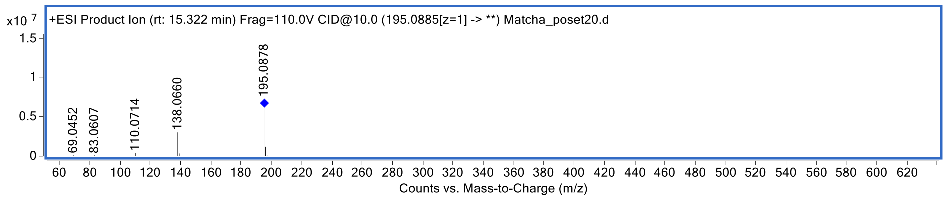
**Caffeoyl-feruloylquinic Acid**	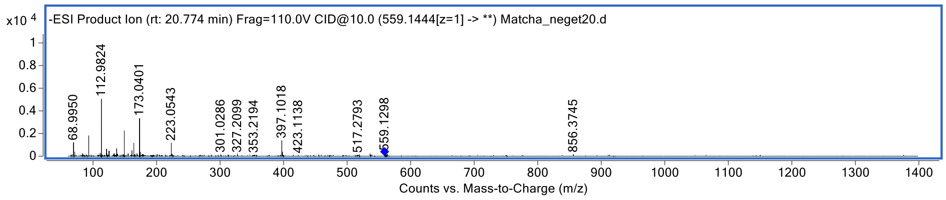
**Coumaroylquinic Acid**	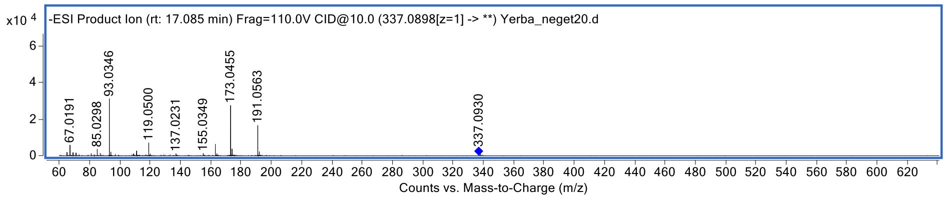

**Epicatechin**	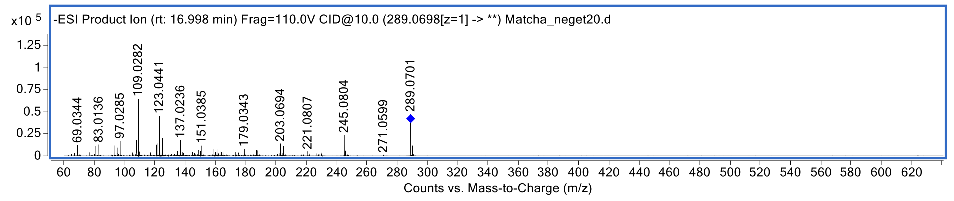
**Epicatechin Gallate**	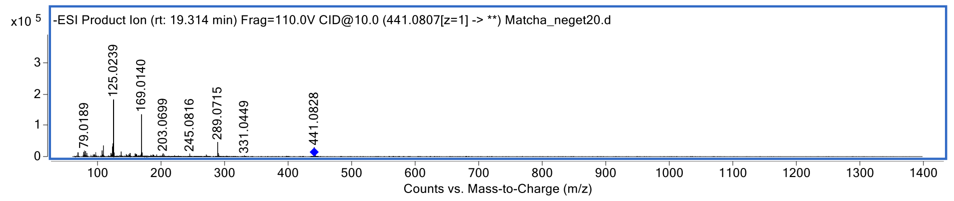
**Epigalocatechin gallate**	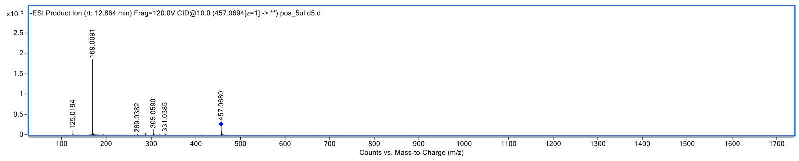
**Feruloylquinic Acid**	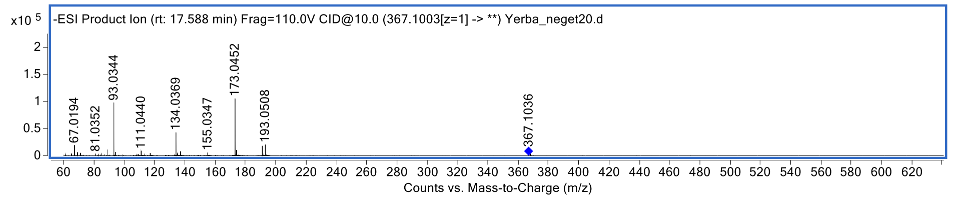
**Gallocatechin**	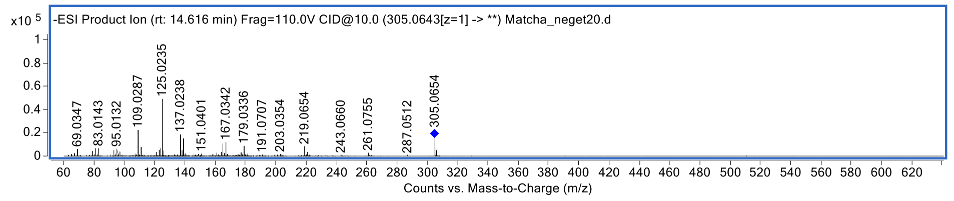
**Gambiriin A1**	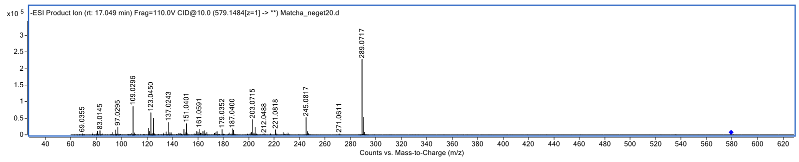

<table>
<tr><td>Isoquercetin</td><td>
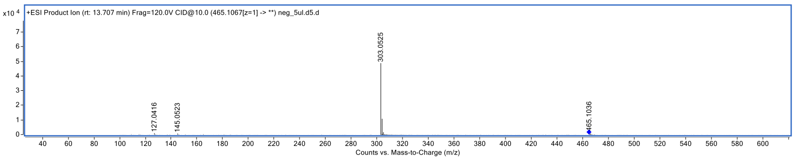

</td></tr>
<tr><td>Kaempferol hexoside</td><td>
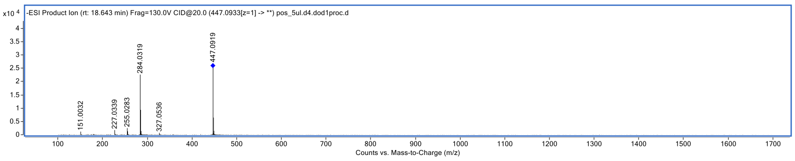

</td></tr>
<tr><td>Kaempferol rhamnodi-glucoside isomer 1</td><td>
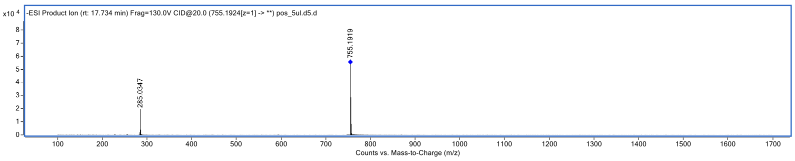

</td></tr>
<tr><td>Kaempferol rhamnodi-glucoside isomer 2</td><td>
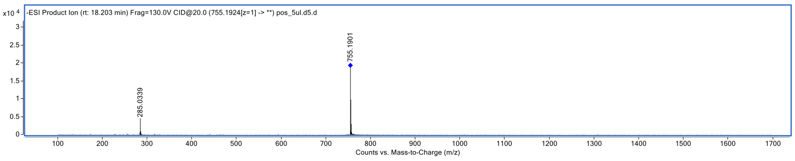

</td></tr>
<tr><td>Kaempferol-O-Galactoside</td><td>
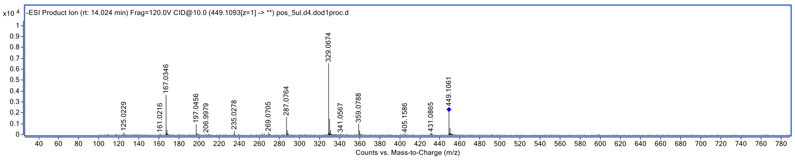

</td></tr>
<tr><td>Quercetin hexoside</td><td>
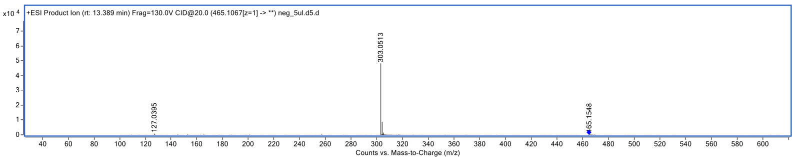

</td></tr>
<tr><td>Quercetin-trihexoside isomer 1</td><td>
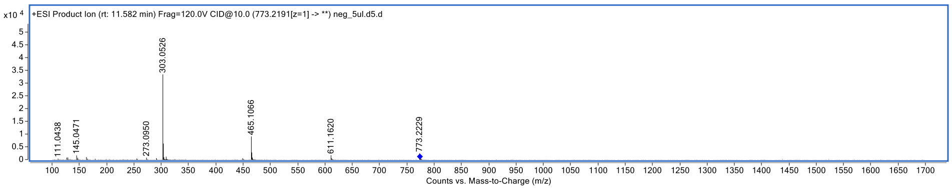

</td></tr>
<tr><td>Quercetin-trihexoside isomer 2</td><td>
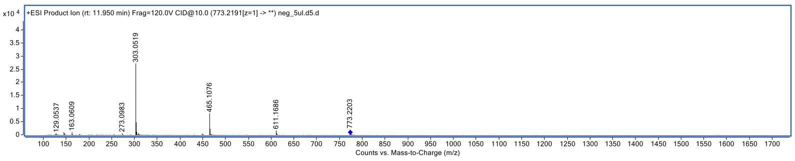

</td></tr>
</table>

**Rutin**	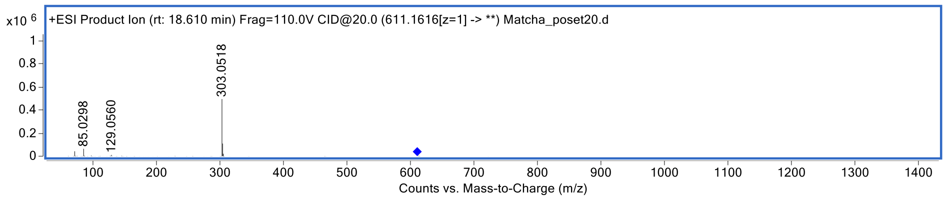
**Theanine**	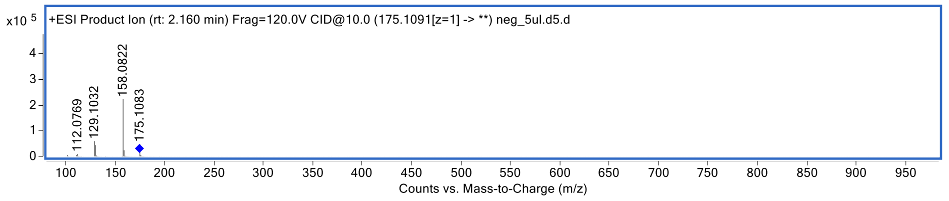
**Theobromine**	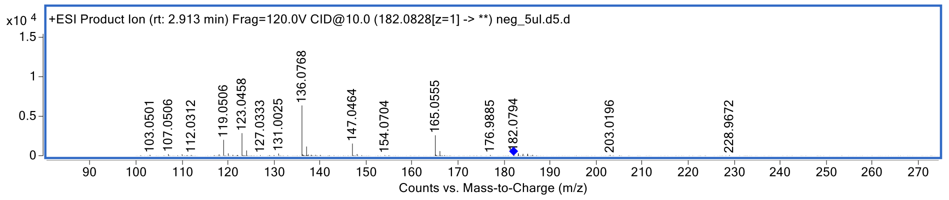
**Theogallin**	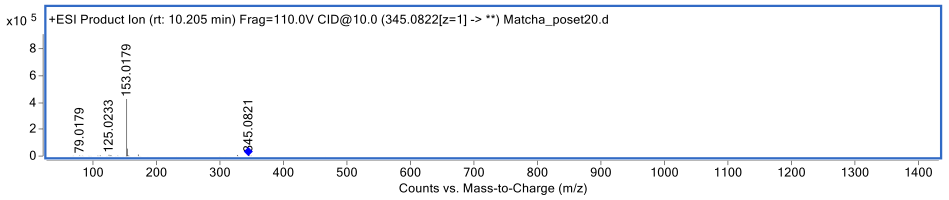
**Tyrosol glucoside**	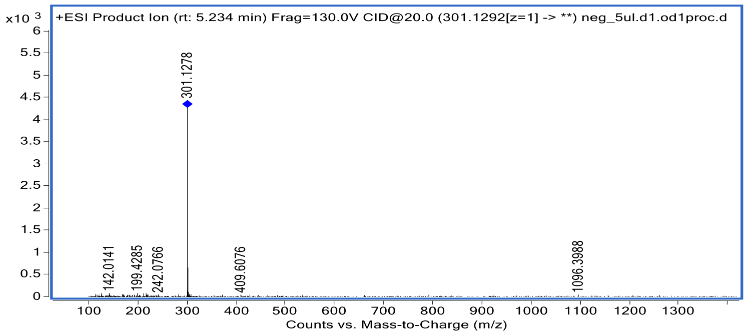
**Vitexin**	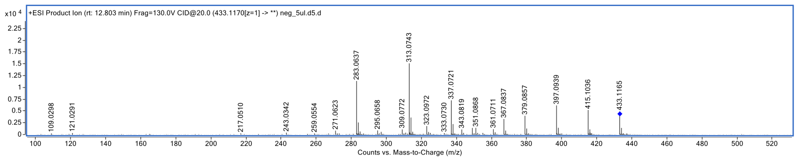

Table S2. Descriptive statistics and Tukey HSD compact letter display within each plant material.

Endpoint	Plant material	Solvent	n	Mean ± SD	Tukey letters
SPF	Matcha	H_2O	3	11.74 ± 0.28	b
SPF	Matcha	EtOH	3	11.50 ± 0.06	b
SPF	Matcha	PG	3	18.54 ± 0.65	a
SPF	Yerba Mate	H_2O	3	27.87 ± 0.24	a
SPF	Yerba Mate	EtOH	3	26.19 ± 4.22	a
SPF	Yerba Mate	PG	3	28.65 ± 0.14	a
DPPH pEC_{50}	Matcha	H_2O	3	1.20 ± 0.14	a
DPPH pEC_{50}	Matcha	EtOH	3	1.42 ± 0.14	a
DPPH pEC_{50}	Matcha	PG	2	0.51 ± 0.14	b
DPPH pEC_{50}	Yerba Mate	H_2O	3	1.17 ± 0.04	a
DPPH pEC_{50}	Yerba Mate	EtOH	3	1.13 ± 0.04	a
DPPH pEC_{50}	Yerba Mate	PG	2	0.18 ± 0.03	b
Tyrosinase inhibition	Matcha	H_2O	3	50.14 ± 1.79	bc
Tyrosinase inhibition	Matcha	EtOH	3	53.02 ± 1.81	ac
Tyrosinase inhibition	Matcha	PG	3	55.65 ± 1.07	a
Tyrosinase inhibition	Yerba Mate	H_2O	3	13.12 ± 1.39	a
Tyrosinase inhibition	Yerba Mate	EtOH	3	−7.87 ± 2.16	c
Tyrosinase inhibition	Yerba Mate	PG	3	5.77 ± 2.85	b
A375 cytotoxicity	Matcha	H_2O	12	60.92 ± 6.38	b
A375 cytotoxicity	Matcha	EtOH	12	80.57 ± 4.42	a
A375 cytotoxicity	Matcha	PG	12	72.77 ± 12.72	a
A375 cytotoxicity	Yerba Mate	H_2O	12	88.49 ± 3.88	a
A375 cytotoxicity	Yerba Mate	EtOH	12	93.90 ± 4.62	a
A375 cytotoxicity	Yerba Mate	PG	12	41.53 ± 18.70	b
Fibroblast cytotoxicity	Matcha	H_2O	12	43.75 ± 12.89	a
Fibroblast cytotoxicity	Matcha	EtOH	12	4.70 ± 5.63	b
Fibroblast cytotoxicity	Matcha	PG	12	12.40 ± 8.82	b
Fibroblast cytotoxicity	Yerba Mate	H_2O	12	43.37 ± 11.15	a
Fibroblast cytotoxicity	Yerba Mate	EtOH	12	4.70 ± 5.63	c
Fibroblast cytotoxicity	Yerba Mate	PG	12	28.92 ± 11.40	b

Note: Values are presented as mean ± SD. Different letters indicate statistically significant differences between solvents within the same plant material and endpoint according to Tukey's HSD test ($p < 0.05$). For DPPH pEC_{50}, higher values indicate stronger radical-scavenging activity. For A375 and fibroblast cytotoxicity, higher values indicate stronger cytotoxicity. Propylene glycol DPPH groups were calculated from n = 2.

Table S3. Two-way ANOVA summary for the effects of plant material, extraction solvent and their interaction.

Endpoint	Plant material	Extraction solvent	Plant material × solvent
SPF	$F(1,12)=127.43$; $p=9.53\times10^{-8}$ ***	$F(2,12)=15.62$; $p=4.57\times10^{-4}$ ***	$F(2,12)=4.83$; $p=0.0289$ *
DPPH pEC_{50}	$F(1,10)=0.10$; $p=0.7641$ ns	$F(2,10)=50.06$; $p=6.18\times10^{-6}$ ***	$F(2,10)=3.79$; $p=0.0596$ ns
Tyrosinase inhibition	$F(1,12)=551.80$; $p=2.12\times10^{-11}$ ***	$F(2,12)=6.11$; $p=0.0148$ *	$F(2,12)=57.47$; $p=7.14\times10^{-7}$ ***
A375 cytotoxicity	$F(1,66)=45.00$; $p=5.34\times10^{-9}$ ***	$F(2,66)=11.59$; $p=4.86\times10^{-5}$ ***	$F(2,66)=55.73$; $p=6.67\times10^{-15}$ ***
Fibroblast cytotoxicity	$F(1,66)=0.01$; $p=0.9236$ ns	$F(2,66)=54.84$; $p=9.31\times10^{-15}$ ***	$F(2,66)=5.97$; $p=0.0041$ **

Note: DPPH activity was analyzed as pEC_{50}; higher values indicate stronger radical-scavenging activity. A375 and fibroblast data represent cytotoxicity; higher values indicate a stronger cytotoxic effect. Significance levels: ns, not significant; * $p < 0.05$; ** $p < 0.01$; *** $p < 0.001$.

Table S4. PCA-derived loadings of phytochemical variables on latent factors F1–F3 used in bioactivity regression models.

No.	Compound	F1	F2	F3
1	Caffeine	0.491	0.765	0.328
2	Caffeoylquinic Acid 1	−0.648	−0.623	−0.258
3	Caffeoylquinic Acid 2	−0.673	−0.349	−0.599
4	Epicatechin	0.180	0.972	0.070
5	Epicatechin Gallate	0.113	0.840	0.288
6	Gallocatechin	0.254	0.944	0.091
7	Kaempferol-O-Galactoside	−0.511	−0.005	−0.855
8	Theanine	0.935	0.294	0.198
9	Theobromine	−0.594	−0.620	−0.402
10	Theogallin	0.988	0.010	0.047
11	Tyrosol-O-Glucoside	−0.188	0.551	0.813
12	Total polyphenols	−0.684	−0.351	−0.564
13	Total flavonoids	−0.877	−0.314	−0.095

Note: The table presents loadings after PCA with VARIMAX rotation. F1–F3 were used as explanatory variables in the regression models reported in the main text and Table S5.

Table S5. Performance parameters of chemometric regression models and leave-one-out cross-validation.

Endpoint	Factors	Model SD	R	D/R²	F	PRESS	Q²LOO	SDEPLOO / RMSECVLOO	MAELOO
Tyrosinase inhibition	F1, F3	0.090	0.997	0.993	608.32	94.66	0.976	3.97	3.22
SPF	F1, F3	0.366	0.945	0.893	33.39	125.40	0.550	4.57	3.52
Fibroblast cytotoxicity	F1, F2	0.146	0.991	0.983	230.96	208.45	0.912	5.89	4.85

Note: R, correlation coefficient; D/R^2, determination coefficient; F, F-test value; PRESS, predicted residual sum of squares; Q^2_LOO, cross-validated determination coefficient; SDEP_LOO, standard deviation error of prediction; RMSECV_LOO, root mean square error of cross-validation; MAE_LOO, mean absolute error. Autoscaling during LOOCV was performed independently within each training subset.

Table S6. Critical wavelength (λc) and UVA/UVB absorbance ratio of matcha (*Camellia sinensis*) and yerba mate (Ilex paraguariensis) extracts prepared in water, 20% (*v*/*v*) ethanol and 50% (*v*/*v*) propylene glycol (PG), calculated from UV absorption spectra recorded at 1 nm intervals between 200 and 400 nm.

Green solvent	Concentration (*v*/*v*)	λc (nm)	UVA/UVB ratio
Matcha (Camellia sinensis) extracts			
H2O	5%	373.3	1.20
H2O	2.5%	373.4	1.21
H2O	1%	375.0	1.34
20% EtOH	5%	371.7	0.83
20% EtOH	2.5%	375.7	0.76
20% EtOH	1%	380.7	0.60
50% PG	5%	373.1	0.97
50% PG	2.5%	374.4	0.82
50% PG	1%	380.4	0.68
Yerba mate (Ilex paraguariensis) extracts			
H2O	5%	359.1	1.54
H2O	2.5%	353.1	1.21
H2O	1%	350.4	1.13
20% EtOH	5%	364.1	1.81
20% EtOH	2.5%	360.4	1.59
20% EtOH	1%	359.8	1.43
50% PG	5%	364.3	1.90
50% PG	2.5%	358.8	1.56
50% PG	1%	358.5	1.53
